# A new approach for balancing the microbial synthesis of ethyl acetate and other volatile metabolites during aerobic bioreactor cultivations

**DOI:** 10.1002/elsc.202000047

**Published:** 2020-12-21

**Authors:** Christian Löser, Christian Kupsch, Thomas Walther, Andreas Hoffmann

**Affiliations:** ^1^ Chair of Bioprocess Engineering, Institute of Natural Materials Technology Technische Universität Dresden Dresden Germany

**Keywords:** acetaldehyde, ethanol, *Kluyveromyces marxianus*, modeling, partition coefficient

## Abstract

Ethyl acetate is an organic solvent with many industrial applications, currently produced by energy‐intensive chemical processes based on fossil carbon resources. Ethyl acetate can be synthesized from renewable sugars by yeasts like *Kluyveromyces marxianus* in aerobic processes. However, ethyl acetate is highly volatile and thus stripped from aerated cultivation systems which complicate the quantification of the produced ester. Synthesis of volatile metabolites is commonly monitored by repeated analysis of metabolite concentrations in both the gas and liquid phase. In this study, a model‐based method for quantifying the synthesis and degradation of volatile metabolites was developed. This quantification of volatiles is solely based on repeatedly measured gas‐phase concentrations and allows calculation of reaction rates and yields in high temporal resolution. Parameters required for these calculations were determined in abiotic stripping tests. The developed method was validated for ethyl acetate, ethanol and acetaldehyde which were synthesized by *K. marxianus* DSM 5422 during an iron‐limited batch cultivation; it was shown that the presented method is more precise and less time‐consuming than the conventional method. The biomass‐specific synthesis rate and the yield of ethyl acetate varied over time and exhibited distinct momentary maxima of 0.50 g g^‒1^h^‒1^ and 0.38 g g^‒1^ at moderate iron limitation.

**Abbreviations**
Eat1: ethanol acetyltransferase 1
VOCs: volatile organic compounds

## 1 INTRODUCTION

Ethyl acetate is easily degraded by bacteria [1, 2] and thus rated as an environmentally friendly organic solvent, possessing versatile industrial applications: as dissolver in chemical reactions, for extraction and chromatographic recovery of active substances, for cleaning surfaces, for processing surface formulations, and for production of adhesives, print colors, paints and herbicide formulations [3, 4]. Ethyl acetate is currently produced from fossil resources by energy‐intensive petrochemical processes [3, 5], although microbial synthesis of this ester from sugar‐rich resources exhibits a high economical potential [6]. Especially yeasts are able to produce this ester in high amounts [3, 7, 8] whereby *Wickerhamomyces anomalus* [8, 9, 10, 11, 12, 13], *Cyberlindnera jadinii* [8, 14−16] and *Kluyveromyces marxianus* [7, 8, 17−27] have turned out as the most promising species. Recent identification of ethanol acetyltransferase 1 (Eat1) as the key enzyme for biosynthesis of ethyl acetate in yeasts [13] and the latest findings regarding the reaction kinetics [13] and localization of this enzyme in the mitochondria [8, 28] enable rational metabolic engineering for developing improved producer strains [29−31].

Evaluation of ethyl acetate production has been usually based on the ester yield ($Y_{EA/S}$) or the pathway efficiency (the $Y_{EA/S}$‐$Y_{EA/S,max}$ ratio) as an average value of the total process. However, there are some more assessment criteria such as the productivity ($R_{EA}$), the biomass‐specific synthesis rate ($r_{EA}$), and the product selectivity, which are of high importance for economic process evaluation, but have not yet received much attention. The productivity $R_{EA}$ informs about the mass of synthesized ethyl acetate per reaction volume and time which allows evaluation of the process effectiveness, while $r_{EA}$ characterizes the efficiency of the biological system. The product selectivity is the mass of the produced target product divided by the mass of all formed products; a high product selectivity is wanted since synthesis of side‐products wastes sugar and by‐products pollute the target product.

Ethyl acetate microbially synthesized in aerated bioreactors is quickly transferred from the cultivation medium to the gas phase and then discharged from the reactor by the exhaust gas [7, 8, 17−19, 22−24, 31, 32] which is called gas stripping. Other co‐produced volatile organic compounds (VOCs) such as ethanol [19, 22, 33−37] and acetaldehyde [38] are stripped in the same manner. Stripping enables an efficient process‐integrated recovery of volatile microbial products [37, 39−41] by saving time and energy, reducing the number of required process stages, avoiding VOC accumulation to inhibitory levels, and reducing the risk of microbial VOC degradation [25, 29, 31, 35, 36].

However, VOC stripping complicates observation of product formation and determination of yields and synthesis rates. Quantification of volatile ethyl acetate in bioreactor experiments was thus often afflicted with deficiencies; stripping was fully ignored [16, 42] or intended retention of stripped ester was incomplete (retention by using condensers [17, 18, 32], decane‐filled trap [12] or adsorbent materials [43] failed). Unnoticed losses of ethyl acetate inevitably result in under‐determined ester synthesis. Stripping of ethyl acetate from aerated cultivation systems can hardly be prevented but calls for adequate handling. Precise balancing of VOC synthesis in aerated bioreactors was hitherto based on regular measurements of the VOC concentrations in the culture medium as well as the exhaust gas [8, 19, 21, 22, 24, 31]. Exclusive quantification of the stripped ester without considering the dissolved ester [44, 45] is insufficient for determining synthesis rates since this strategy ignores the temporal accumulation of already synthesized but not yet stripped ethyl acetate. Unfortunately, the quantification of liquid‐phase concentrations is time‐consuming, labor‐intensive and less precise than the quantification of gas‐phase concentrations (due to the heavy dilution of the sugar‐rich and thus highly viscous medium samples).

**PRACTICAL APPLICATION**

Ethyl acetate is an environmentally friendly solvent with many applications. This ester is produced by several yeast species from renewable sugar. Ethyl acetate is highly volatile which enables a process‐integrated product recovery by stripping but also complicates quantification of ester synthesis during process development and optimization. Here, we present a new approach for model‐based balancing the ester synthesis solely based on ester concentrations measured in the exhaust gas. The proposed approach delivers rates and yields of ethyl acetate and other volatile metabolites in a high temporal resolution which can be further used to evaluate and control fermentation processes. The practicability of the new method is demonstrated for an example.

VOC balancing could be significantly simplified by analyzing the VOC only in one phase and making use of a constant ratio between the VOC concentrations in the culture medium and headspace gas. Such a correlation of the two VOC concentrations was constantly observed for ethyl acetate during bioreactor experiments [7, 19, 22−24]. This ratio is identical with the partition coefficient, provided that the two phases are nearly equilibrated which is at least true for well‐mixed systems like stirred bioreactors [3, 19]. The proposed balancing method was already successfully applied to processes with ester synthesis in sealed non‐aerated cultivation bottles [21, 25].

The partition coefficient as an important parameter for this ester balancing depends on many factors such as the considered VOC [7, 46−48], the temperature [7, 19, 23, 35, 48], and the presence of other dissolved compounds like minerals [49−52], sugars [19, 53, 54] and other organic substances [55, 56]. This means that the partition coefficient in a real culture medium/air system can significantly differ from the partition coefficient in a water/air system [7, 19]. Real partition coefficients of VOCs have therefore to be determined individually for each specific culture medium. Furthermore, some VOC retention by the condenser plays also a role and results in a slowed down stripping process [33, 34] which again interferes with VOC balancing. This retention effect is compound‐specific and seems to depend on the condenser design and operation [33] so that this subject requires specific consideration for each individual cultivation system.

In this study, we report on analysis of the synthesis of ethyl acetate and other VOCs in aerated bioreactors. Quantification of VOCs is here exclusively based on periodically measured VOC concentrations in the exhaust gas of the bioreactor. The new model‐based quantification method allows for the calculation of time‐dependent yields, synthesis rates and cumulative masses of VOCs in a high temporal resolution. This high temporal resolution allows the observation of dynamically changing synthesis rates, e.g., the detection of short but highly productive synthesis phases as well as transition from VOC synthesis to VOC reutilization. The method requires partition coefficients and retention efficiencies for relevant VOCs under the applied conditions (a given culture medium, temperature, condenser system and so on) which were simply and precisely determined by abiotic stripping experiments. Finally, the feasibility of this innovative approach is demonstrated for the three volatiles ethyl acetate, ethanol and acetaldehyde synthesized by *K. marxianus* DSM 5422 from glucose during an aerobic iron‐limited batch cultivation in a stirred bioreactor.

## 2 MATERIALS AND METHODS

### 2.1 Microorganism and culture medium

The yeast *Kluyveromyces marxianus* DSM 5422 originates from the Deutsche Sammlung von Mikroorganismen und Zellkulturen GmbH (Braunschweig, Germany). Inocula were obtained by one‐day cultivation on yeast extract‐glucose‐chloroamphenicol agar (YGC agar, Roth GmbH, Germany) at 32°C.

The culture medium consisted of a glucose‐based mineral medium. For preparing 1 L medium, an autoclaved 1‐L Schott bottle was filled with 250 mL mineral solution, 250 mL glucose stock solution, 2 mL vitamin solution and 0.2 mL trace‐element solution and then filled up to 1 L with sterile water using a balance. Mineral solution was prepared by dissolving 20 g (NH_4_)_2_SO_4_, 12 g KH_2_PO_4_, 2 g MgSO_4_· H_2_O, 0.4 g NaCl and 4 mg CaCl_2_· H_2_O separately in water, the single solutions were combined and the received mixture was filled up with water to a volume of 1 L. The mineral solution was heat‐sterilized for 20 min at 121°C. Glucose stock solution contained 80 g L^−1^ glucose (Carl Roth GmbH; iron content ≤5 μg g^−1^) in water and was heat‐sterilized for 20 min at 121°C. The vitamin solution is similar to those of Wickerham [57] and Postma et al. [58] and was prepared as follows: all compounds were dissolved separately in 20 mM NaOH, namely 12.5 g inositol in 200 mL, but 500 mg Calcium pantothenate, 500 mg nicotinic acid, 500 mg thiamine·HCl, 500 mg pyridoxine·HCl, 100 mg 4‐aminobenzoate, 100 mg riboflavin, 100 mg folic acid, and 25 mg biotin in 100 mL each. The single solutions were combined yielding 1 L vitamin solution of pH 8 which was filter‐sterilized (regenerated‐cellulose filter, pore size 0.45 μm, Sartorius Stedim Biotech GmbH, Göttingen). Trace‐element solution was composed like normative trace‐element solution of Urit et al. [20]. A modified trace‐element solution was used in iron‐limitation experiments where iron sulfate had been omitted. The trace‐element solutions were heat‐sterilized for 20 min at 121°C.

### 2.2 Bioreactor cultivation

All cultivations were conducted in a 1‐L stirred bioreactor, mixed by three six‐bladed Rushton turbines and controlled by an ADI 1030 Biocontroller (Applikon) as described previously [19]. The reactor was charged with 0.3 mL Antifoam A (Fluka), autoclaved for 20 min at 121°C, and its interior dried by a sterile air flow. The reactor was filled with 600 mL sterile medium using a peristaltic pump and balance (medium density was 1008.3 g L^−1^). The pH of the medium was adjusted to 6.0 by pumping a small amount of 2 M NaOH into the reactor. The reactor was operated at a temperature of 32°C, a stirrer speed of 1200 rpm, and an air flow of 30 L h^−1^ (given for 0°C and 101325 Pa; water content 0.008 L L^−1^). For inoculation, one loop of biomass from a plate culture was suspended in 2 mL sterile water, and 0.15 mL of this suspension was injected into the bioreactor. The pH was controlled to ≥5 by supply of 2 M NaOH. Samples of 2.4 mL cell suspension were repeatedly withdrawn; 0.4 mL were used for $OD_{600nm}$ measurements and 2.0 mL were processed according to [19] for determining the biomass dry weight and concentrations of dissolved compounds. Final sampling of 4 × 40 mL suspension allowed precise biomass analysis. Volatile organic compounds (VOCs) in the exhaust gas were analyzed in 15‐min intervals as described by Urit et al. [19]. The O_2_ and CO_2_ content of the exhaust gas were continuously measured by an EL3020 gas analyzer (ABB, Germany).

### 2.3 Stripping experiments

Stripping of VOCs from the aerated bioreactor was performed as described earlier [19, 34] with the modification that the temporal change of the VOC concentration was analyzed in the gas rather than the liquid phase. The stripping experiments were performed like a cultivation experiment (600 mL medium of pH 6, 32°C, stirring with 1200 rpm, and aeration with 30 L h^−1^), but there were also some differences. The culture medium was supplemented with 1 g L^−1^ sodium azide to avoid microbial contamination during long‐term stripping. The bioreactor was operated with or without a condenser. Water losses by evaporation were avoided by moistening the supplied air flow at a suited temperature (at 12.5°C during stripping with condenser, or at 32°C during stripping without condenser). In stripping experiments without condenser, the bioreactor was completely insulated by polystyrene‐foam flakes to avoid condensation of water in the headspace of the bioreactor. The DO sensor was substituted by a PTFE‐covered silicon septum. The sterile medium was not inoculated but spiked with 1.8 g VOC through the septum using a syringe. Then, aeration was started and the gas phase was repeatedly sampled (the headspace gas via the septum, and the exhaust gas at the gas‐line exit) and analyzed regarding the VOC content by gas chromatography.

### 2.4 Analyses

The $OD_{600nm}$ of cell suspensions was measured by a DU 520 photometer (Beckman). Biomass dry weights were determined by separating yeast cells from the suspension via centrifugation, washing the pellet twice with deionized water and drying at 105°C until weight constancy. Sugar was determined by the DNS method according to Hortsch et al. [59]. VOCs in the liquid and gas phase were analyzed by gas chromatography (GC) as described in [19].

## 3 RESULTS AND DISCUSSION

Balancing of microbial synthesis of ethyl acetate was so far based on ester concentrations measured in both the liquid and gas phase [3, 8, 19, 20−22, 24, 31]. Here, a new balancing method was developed which is solely based on gas‐phase concentrations. This alternative method offers several advantages: (1) exhaust‐gas sampling does not affect the process, although liquid‐phase sampling for sugar and biomass analyses still takes some influence; (2) GC analysis of gas samples is more precise and less time‐consuming compared to liquid‐sample analysis; (3) quicker GC analysis enables a higher time resolution of measurements and balancing. The new method thus enables the calculation of synthesis rates, masses and yields of formed VOCs in a high temporal resolution.

The new balancing method requires two parameters: (1) the partition coefficient of the considered VOC in a given liquid/gas system; (2) the retention efficiency of the condenser of bioreactor for this VOC. Both parameters were determined in high precision via abiotic stripping experiments performed with the culture medium/air system.

The new mode of model‐based data processing was demonstrated on the example of the volatile ethyl acetate, ethanol and acetaldehyde synthesized by *K. marxianus* DSM 5422 from glucose under iron‐limited conditions. The synthesis rates, synthesized masses and yields of these VOCs were calculated for 15‐min intervals. The high temporal resolution of VOC balancing allowed a deeper insight into the process of ester synthesis since earlier studies demonstrated [19, 20, 22−24] that the rate of ester synthesis changes quickly during the cultivation process. A future large‐scale production process will be aimed at maintaining a high rate of ethyl acetate synthesis via process control which requires the determination of the changing rate with a sufficiently high resolution. Another benefit of the high resolution is that a switch from VOC synthesis to a possible VOC degradation is detected immediately in case the gas‐phase concentration of the VOC is measured quasi‐online via a mass spectrometer.

### 3.1 Balancing the synthesis of volatile metabolites

Superimposition of synthesis/degradation and stripping has already been modeled for ethanol [34] and ethyl acetate [19]. The temporal change of the VOC concentration of the liquid phase in an aerated stirred bioreactor with exhaust‐gas condenser operated as batch process is described as follows:

(1) $$\frac{dC_{VOC,L}}{dt}=R_{VOC}\;-C_{VOC,L}\cdot \frac{1-\beta_{VOC}}{\frac{1}{k_{VOC,L}a}+\frac{K_{VOC,L/G}}{\left(F_{G,R}/V_{L}\right)}}$$

The $R_{VOC}$ parameter stands for the volume‐specific reaction rate, $k_{VOC,L}a$ and $K_{VOC,L/G}$ symbol the phase‐transfer coefficient and the partition coefficient of the considered VOC, respectively, the $F_{G,R}$‐$V_{L}$ ratio is the specific gas‐flow rate leaving the bioreactor, and $\beta_{VOC}$ is the efficiency of the VOC retention by the condenser [34]. In detail, part of the gaseous VOC is absorbed by condensing water and then transported back to the bioreactor dissolved in the condensate. $\beta_{VOC}$ represents the mass flow of VOC transported back to the reactor related to mass flow of VOC entering the condenser with the gas phase.

Stripping of a dissolved VOC is a two‐step process where the VOC is at first transported from the liquid to the headspace gas (determined by $k_{VOC,L}a$ and $K_{VOC,L/G}$) and, in a second step, the gaseous VOC is discharged from the headspace with the gas flow $F_{G,R}$. At intensive stirring, the $k_{VOC,L}a$ value is distinctly larger than the $F_{G,R}$/$V_{L}$‐$K_{VOC,L/G}$ ratio so that the $1/k_{VOC,L}a$ term in Equation (1) becomes negligible, resulting in a simplified equation:

(2) $$\begin{array}{ccc} \frac{dC_{\mathrm{VOC},L}}{dt} & = & R_{\mathrm{VOC}}\;-C_{\mathrm{VOC},L}\cdot \frac{1-\beta_{\mathrm{VOC}}}{K_{\mathrm{VOC},L/G}}\cdot \frac{F_{G,R}}{V_{L}}\\ & & \mathrm{at}\;k_{\mathrm{VOC},L}a\gg \frac{F_{G,R}/V_{L}}{K_{\mathrm{VOC},L/G}} \end{array}$$

Stripping experiments clearly demonstrated that this simplification is permissible at least for ethyl acetate and ethanol even if the stirring speed is as low as 100 rpm [19]. For substances with a low partition coefficient such as styrene [40] and toluene [60], this simplification is not applicable.

Balancing the VOCs for the considered process based on measured gas‐phase concentrations requires substitution of $C_{VOC,L}$ by the gas‐phase concentration. Intense mixing in the stirred bioreactor results in an approximate phase equilibrium:

(3) $$\frac{C_{VOC,L}}{C_{VOC,G,R}}=K_{VOC,L/G}\;\mathrm{at}\;\mathrm{intense}\;\mathrm{mixing}$$

Substitution of $C_{VOC,L}$ in Equation (2) by Equation (3) and rearrangement yields (minimal temporal $K_{VOC,L/G}$ variations neglected):

(4) $$\begin{array}{ccc} \frac{dC_{VOC,G,R}}{dt} & = & \frac{R_{VOC}}{K_{VOC,L/G}}\\ & & -\;C_{VOC,G,R}\cdot \frac{1-\beta_{VOC}}{K_{VOC,L/G}}\cdot \frac{F_{G,R}}{V_{L}} \end{array}$$

This equation enables calculation of the reaction rate $R_{VOC}$ from repeatedly measured headspace concentrations of the VOC ($C_{VOC,G,R}$) provided that $K_{VOC,L/G}$ is known. In cultivation experiments, the gas‐phase content of VOCs was measured at the exit of the exhaust‐gas line ($C_{VOC,G}$) rather than in the headspace. Therefore, the measured $C_{VOC,G}$ data have to be converted into $C_{VOC,G,R}$ data.

The VOC retention by the condenser and volumetric effects (partial dehumidification and changes in temperature modify the gas flow) impact the VOC concentration in the gas flow. Balancing the mass flow of VOC allows to formulate a relation between $C_{VOC,G,R}$ and $C_{VOC,G}$:

(5) $$C_{VOC,G,R}\cdot F_{G,R}\cdot \left(1-\beta_{VOC}\right)\;=C_{VOC,G}\;\cdot F_{G}$$

This means, there exists a constant relation between both VOC concentrations depending on the $F_{G}$‐$F_{G,R}$ ratio and $\beta_{VOC}$. Equation (5) is used for substituting the unknown $C_{VOC,G,R}$ concentration in Equation (4) by the measured $C_{VOC,G}$ values:

(6) $$\frac{dC_{VOC,G}}{dt}=\left(R_{VOC}-C_{VOC,G}\cdot \frac{F_{G}}{V_{L}}\right)\;\cdot \frac{1-\beta_{VOC}}{K_{VOC,L/G}}\cdot \frac{F_{G,R}}{F_{G}}$$

The unknown gas flow $F_{G,R}$ is calculated from $F_{G}$, taking flow changes by partial dehumidification and thermal effects into account (the loss in pressure and the flow reduction by VOC retention are insignificant):

(7) $$F_{G,R}=F_{G}\;\cdot \frac{T_{G,R}}{T_{G}}\cdot \frac{1-x_{W,G}}{1-x_{W,G,R}}$$

Neither the temperatures nor the water contents varied over time ($T_{G,R}$ = 305.15 K, $T_{G}$ = 298.15 K, $x_{W,G,R}$ = 0.0469 L L^−1^, and $x_{W,G}$ = 0.0143 L L^−1^ in the cultivation experiments shown below) so that the $F_{G,R}$‐$F_{G}$ ratio is constant, namely $F_{G,R}$/$F_{G}$ = 1.0585. The flow at the gas‐line exit $F_{G}$ is calculated from the supplied gas flow $F_{G,0}$ according to Duboc et von Stockar [33], taking changes of the gas composition into account. These changes result from the bioprocess (O_2_ consumption and CO_2_ formation), moistening in the reactor, and dehumidification in the condenser:

(8) $$F_{G}=F_{G,0}\;\cdot \frac{T_{G}}{T_{G,0}}\cdot \frac{1-x_{O_{2},G,0}-x_{CO_{2},G,0}-x_{W,G,0}}{1-x_{O_{2},G}-x_{CO_{2},G}-x_{W,G}}$$

The bioreactor was aerated with a defined air flow using a mass‐flow controller ($F_{G,0}$ = 30 L h^−1^ at $p_{G,0}$ = 101325 Pa, $T_{G,0}$ = 273.15 K, $x_{O_{2},G,0}$ = 0.2078 L L^−1^, $x_{CO_{2},G,0}\;$= 0.0004 L L^−1^, and $x_{W,G,0}$ = 0.008 L L^−1^) while the $x_{O_{2},G}$ and $x_{CO_{2},G}$ values were continuously measured. Evaporating VOCs change more or less the gas composition and thus increase the gas flow. The degree of such an $F_{G}$ increase is determined by the VOC concentration in the gas phase, $C_{VOC,G}$. The effect of $C_{VOC,G}$ on the gas flow can be taken into account by extending Equation (8) (for details see references [22] and [33]). In the presented cultivations, the ester synthesis was limited by the quite low sugar content of the medium so that the concentration of volatiles in the gas phase was relatively small and the increase of the gas flow was less than 0.5% and therefore neglected.

The following equation is used to convert measured gas‐phase concentrations into required liquid‐phase concentrations (for details see Supporting Information 1):

(9) $$C_{VOC,L}=C_{VOC,G}\;\cdot \frac{T_{G}}{T_{G,R}}\cdot \frac{1-x_{W,G,R}}{1-x_{W,G}}\cdot \frac{K_{VOC,L/G}}{1-\beta_{VOC}}$$

The differential of Equation (6) was converted into a difference and the obtained equation was rearranged to give:

(10) $$\begin{array}{ccc} & & R_{VOC}\left(t\ldots t+\Delta t\right)=\frac{C_{VOC,G}\left(t+\Delta t\right)-C_{VOC,G}\left(t\right)}{\Delta t}\;\cdot \\ & & \frac{K_{VOC,L/G}}{1-\beta_{VOC}}\cdot \frac{F_{G}}{F_{G,R}}+C_{VOC,G}\left(t\ldots t+\Delta t\right)\cdot \frac{F_{G}}{V_{L}} \end{array}$$

$R_{VOC}$ is a process‐related parameter which expresses the mass of formed or consumed VOC per hour and liter liquid volume. This parameter is transformed into a biomass‐specific rate:

(11) $$r_{VOC}\left(t\ldots t+\Delta t\right)=\frac{R_{VOC}\left(t\ldots t+\Delta t\right)}{C_{X}\left(t\ldots t+\Delta t\right)}\;$$

$r_{VOC}$ is a biological parameter characterizing the microbiological system, indicating the mass of synthesized or consumed VOC per hour by one gram of biomass (dry weight). Positive $r_{VOC}$ values mean VOC synthesis, while negative $r_{VOC}$ values stand for VOC utilization.

The cumulative mass of formed VOC till a given time t is the mass of the stripped VOC till the considered moment plus the VOC dissolved in the liquid phase (already synthesized but not yet stripped [19]):

(12) $$m_{VOC}\left(t\right)\;=\int_{\tau =0}^{\tau =t}C_{VOC,G}\left(\tau \right)\cdot F_{G}\cdot d\tau \;+C_{VOC,L}\left(t\right)\cdot V_{L}$$

Substitution of $C_{VOC,L}$ by $C_{VOC,G}$ using Equations (3) and (5) results in:

(13) $$\begin{array}{ccc} m_{VOC}\left(t\right) & = & \int_{\tau =0}^{\tau =t}C_{VOC,G}\left(\tau \right)\cdot F_{G}\cdot d\tau \;\\ & & +\;C_{VOC,G}\left(t\right)\cdot \frac{K_{VOC,L/G}}{1-\beta_{VOC}}\cdot \frac{F_{G}}{F_{G,R}}\cdot V_{L} \end{array}$$

$m_{VOC}\left(t\right)$ steadily increases in time till the moment where the VOC synthesis stops. $m_{VOC}\left(t\right)$ can even diminish in time in case of a microbial degradation of the dissolved VOC by the cultivated yeasts.

Yields are usually calculated as averages of the total process. The following equation enables to calculate yields of VOCs for time intervals:

(14) $$Y_{VOC/S}\left(t\ldots t+\Delta t\right)=-\frac{R_{VOC}\left(t\ldots t+\Delta t\right)}{R_{S}\left(t\ldots t+\Delta t\right)}$$

This $Y_{VOC/S}\left(t\ldots t+\Delta t\right)$ variable allows to evaluate changes of the yield over time depending on other process variables. $R_{S}\left(t\ldots t+\Delta t\right)$ stands for the sugar consumption rate in the considered time interval and is calculated from the temporal change of the sugar concentration:

(15) $$R_{S}\left(t\ldots t+\Delta t\right)=\frac{C_{S}\left(t+\Delta t\right)-C_{S}\left(t\right)}{\Delta t}$$

$R_{VOC}$ was calculated with Equation (10) for 15‐min intervals ($C_{VOC,G}$ was measurement every 15 min). The cell and sugar concentrations were analyzed in 1‐h intervals but are required in 15‐min intervals for the $r_{VOC}$ and $R_{S}$ calculation by Equations (11) and (15). Therefore, the measured $C_{X}\left(t\right)$ and $C_{S}\left(t\right)$ data were interpolated using a cubic spline smoothing procedure (p=0.95, MATLAB, Curve Fitting Toolbox). Moreover, the adverse volume effect of pH regulation and evaporation of water during the process on the liquid volume ($V_{L}$) and on the $C_{S}$ and $C_{X}$ data was taken into account as explained in [61]. All confidence intervals of data were calculated for a confidence level of 95%.

### 3.2 Characterization of VOC stripping

As seen from the above‐derived Equations (10) to (14), parameters $K_{VOC,L/G}$ and $\beta_{VOC}$ are required for calculating time‐dependent rates, yields and masses of VOCs when the calculation is solely based on repeatedly measured $C_{VOC,G}$ data. Two major methods for determining $K_{VOC,L/G}$ are in use: (1) quantification of the VOC partitioning in an equilibrated liquid/gas system by analyzing the VOC in both phases [47, 62−64] or only in the gas phase [7, 19, 49, 51, 65−69] (then, VOC reduction in the liquid due to evaporation neglected [49, 51, 65, 67, 68] or taken into account by calculation [7, 19, 66, 69]); (2) bubble‐column technique with quantifying the $K_{VOC,L/G}$‐dependent stripping of the dissolved VOC from aerated systems [19, 34, 50, 55, 65, 67, 70]. Comparative studies demonstrated that both methods give the same results [19, 65]. However, the bubble‐column technique is the most reliable procedure since determination of $K_{VOC,L/G}$ is based on relative changes of the VOC concentration in only one phase. Relative changes are precise even if the measured absolute concentrations are erroneous, e.g. by inaccurate calibration. Moreover, determination of VOC retention by a condenser is only possible by stripping experiments carried out at real process conditions.

#### 3.2.1 Stripping rate $k_{VOC}$

Stripping means that a volatile compound is discharged from a liquid by a gas flow passing through this liquid. Here, volatiles like ethyl acetate, acetaldehyde and ethanol are discharged from a culture medium in an aerated bioreactor. The stripping is observed by repeated measurement of the VOC concentration in the headspace and/or in the exhaust gas. Stripping without a microbial process ($R_{VOC}$ = 0) modifies Equations (4) and (6) as follows:

(16) $$\begin{array}{ccc} \frac{d\ln\left(C_{\mathrm{VOC},G,R}\right)}{dt} & = & \frac{d\ln\left(C_{\mathrm{VOC},G}\right)}{dt}=-\frac{1-\beta_{\mathrm{VOC}}}{K_{\mathrm{VOC},L/G}}\cdot \frac{F_{G,R}}{V_{L}}\\ & & =k_{\mathrm{VOC}}\;\mathrm{at}\;R_{\mathrm{VOC}}=0 \end{array}$$

Both differentials represent the specific stripping rate ($k_{VOC}$) which is obtained from repeatedly measured $C_{VOC,G,R}$ or $C_{VOC,G}$ values. After Equation (16), the stripping rate depends on the specific gas‐flow rate ($F_{G,R}$/$V_{L}$) and on the partition coefficient ($K_{VOC,L/G}$) and retention efficiency of the condenser for the studied volatile ($\beta_{VOC}$). The observed stripping rates are used for calculating the wanted parameters $K_{VOC,L/G}$ and $\beta_{VOC}$.

In each stripping experiment, the bioreactor was filled with 0.6 L culture medium, the medium was supplemented with 1.8 g VOC resulting in a liquid‐phase concentration of about 3 g L^−1^, the system was maintained at 32°C, and the stripping process was started by aeration with a gas flow of 30 L h^−1^ air under standard conditions (details given in Section 2.3).

In experiments with a condenser, the condensing water absorbs some VOC and slows down the stripping ($\beta_{VOC}$ > 0), while in the experiments without a condenser, such VOC retention does not occur ($\beta_{VOC}$ = 0). For more details in this respect, it is referred to Urit et al. [19] and Supporting Information 3. The presence or absence of a condenser thus modifies Equation (16). The stripping rate received without a condenser is used to calculate $K_{VOC,L/G}$, while the stripping rate obtained with a condenser allows determination of $\beta_{VOC}$, based on the already known $K_{VOC,L/G}$ value. The complete thermal insulation of the bioreactor is of highest importance since condensation of water in the headspace of the bioreactor slows down the stripping and results in overestimated $K_{VOC,L/G}$ values. Another pitfall concerns losses of water by evaporation; such water losses affect the stripping kinetics by increasing both the specific gas flow and the VOC concentration in the liquid (demonstrated by model simulations based on the above‐given equations as well as verified experimentally). Water losses were effectively avoided by saturating the supplied air with water at 32°C (stripping without condenser) or 12.5°C (stripping with condenser).

**FIGURE 1:**
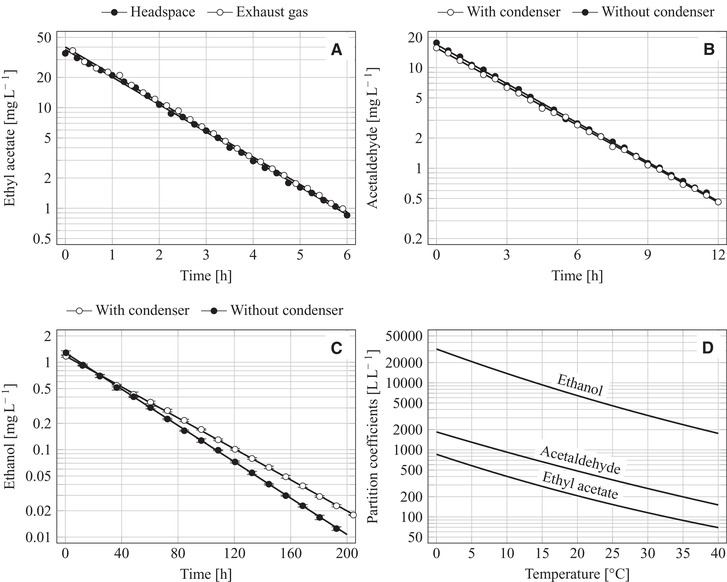
Concentration of volatile organic compounds in the gas phase during the stripping from a 1‐L stirred bioreactor using 0.6 L glucose‐based mineral medium (stirring with 1200 rpm at 32°C and aeration with 30 L h^−1^ under standard conditions); (A) Stripping of ethyl acetate from the reactor fitted without a condenser; (B) Stripping of acetaldehyde from the reactor with or without a condenser; (C) Stripping of ethanol from the reactor with or without a condenser; (D) Temperature‐dependent partition coefficients of VOCs in a water/air system based on data from literature (see Supporting Information 2)

The temporal decline of the measured VOC concentrations followed an exponential function so that the ln($C_{VOC,G}$)‐t and ln($C_{VOC,G,R}$)‐t plots exhibit a linear course (Figure 1) as predicted by Equations (4) and (6). The stripping rates were determined by a linear fit to these logarithmic plots.

The stripping of ethyl acetate was only studied without a condenser since previous tests demonstrated that its stripping is not markedly influenced by the condenser [19]. The logarithmic plots of the headspace and exhaust‐gas concentrations run parallel as expected (Figure 1A) and exhibit a nearly identical stripping rate of $k_{EA}$ = −0.6299 ± 0.0090 h^−1^ (headspace data) and −0.6294 ± 0.0085 h^−1^ (exhaust‐gas data). The distance between both graphs represents a constant $C_{EA,G}$‐$C_{EA,G,R}$ ratio of 1.0580 which conforms to the $F_{G,R}$‐$F_{G}$ ratio of 1.0585 (after Equation (7)).

Stripping of acetaldehyde started with lower gas concentrations (Figure 1B) compared to the initial ester concentration (Figure 1A) despite a uniform initial liquid content, and the removal of a certain proportion of acetaldehyde lasted distinctly longer than the removal of the same proportion of ethyl acetate, which is explained by the lower volatility of acetaldehyde compared to ethyl acetate. The measured acetaldehyde concentrations resulted in the following stripping rates: $k_{AA}^{without\;c.}$ = −0.2989 ± 0.0031 h^−1^, $k_{AA}^{with\;c.}$ = −0.2945 ± 0.0030 h^−1^. Stripping without a condenser was a little quicker than stripping with a condenser due to some retention of acetaldehyde by condensing water.

The stripping of ethanol was significantly slower (Figure 1C) compared to the stripping of the two other studied VOCs. This slower stripping correlates with the low ethanol content in the gas phase; at the start of stripping, the gaseous ethanol concentration was only a little higher than 1 mg L^‒1^. The stripping of ethanol was distinctly influenced by the presence or absence of the condenser (Figure 1C). Stripping without condenser was performed twice resulting in nearly identical rates (only one of these two tests is shown in Figure 1C): $k_{EtOH}^{without\;c.}$ = −0.02394 ± 0.00023 h^−1^ and −0.02389 ± 0.00017 h^−1^. The stripping with the condenser was slower: $k_{EtOH}^{with\;c.}$ = −0.02041 ± 0.00022 h^−1^.

The stripping rates were clearly influenced by physicochemical properties of the three studied VOCs which are connected with their volatility and phase partitioning such as the vapor pressure, the solubility in water and the $K_{O/W}$ coefficient (Table 1). The stripping becomes quicker at a higher $K_{O/W}$ coefficient, a higher vapor pressure and a lower water solubility. The quite hydrophobic ethyl acetate (with the highest $K_{O/W}$ and a limited solubility in water) was quickest stripped, while the highly hydrophilic ethanol (with the lowest $K_{O/W}$ and a non‐limited miscibility with water) was slowest stripped. Acetaldehyde possesses the highest vapor pressure at 32°C, but the limitless miscibility with water counteracts a fast evaporation. Moreover, acetaldehyde is partially hydrated in aqueous solutions [68, 70] which reduces its vaporizable amount and slows down the stripping as well (details below). In this connection, it is referred to Kruis et al. [29] where physicochemical parameters of diverse alkanols, carboxylic acids and esters were correlated with their volatility, toxicity and feasible removal from the cultivation system via gas stripping.

**TABLE 1:** Stripping rates and partition coefficients of ethyl acetate, acetaldehyde and ethanol for describing the distribution between the gas phase and the culture medium at 32°C, together with diverse physicochemical parameters which relate to the partition coefficient; The $K_{\mathrm{VOC},L/G}$ and $\beta_{\mathrm{VOC}}$ parameters were determined in stripping experiments using culture medium as the liquid phase (n. d. = not determined)

	Volatile organic compound		
Parameter	Ethyl acetate	Acetaldehyde	Ethanol
Boiling point at 101325 Pa [°C]	77^a^	21^b^	78^a^
Saturation vapor pressure at 32°C [Pa]	17560^a^	154460^b^	11680^a^
Solubility in water at 32°C [g L^−1^]	67.6^c^	Miscible	Miscible
Octanol/water partition coefficient $K_{O/W}$ at 25°C [L L^−1^]	5.37^a^	2.33^b^	0.048^a^
Partition coefficient $K_{VOC,L/G}$ in air/water at 32°C [L L^−1^]	103.5^c^	236^c^	2864^c^
Stripping rate without a condenser $k_{VOC}^{without\;c.}$ ^d^ [h^−1^]	−0.6297	−0.2989	−0.02392
Stripping rate with a condenser $k_{VOC}^{with\;c.}$ ^d^ [h^−1^]	n. d. ^e^	−0.2945	−0.02041
Partition coefficient $K_{VOC,L/G}$ in air/medium at 32°C [L L^−1^]	92.3 ± 1.3	194.5 ± 2	2430 ± 17
$K_{VOC,L/G}$(Medium)‐$K_{VOC,L/G}$(Water) ratio at 32°C [−]	0.892	0.824	0.849
Condenser retention efficiency $\beta_{VOC}$ (measured) ^f^ [−]	≈0	0.0146	0.147
Condenser retention efficiency $\beta_{VOC}$ (estimated) ^g^ [−]	0.005	0.012	0.142

^a^ Data were taken from [71].

^b^ Data were taken from the NIST database (https://webbook.nist.gov/chemistry/).

^c^ Data were taken from the literature (Figure 1D; for details see Supporting Information 2).

^d^ Stripping conditions are given in the legend of Figure 1 and are described in detail in Section 2.3.

^e^ Former stripping experiments demonstrated that retention of ethyl acetate by a condenser is negligible [19]. Hence, there was no requirement to perform a stripping experiment for ethyl acetate with a condenser.

^f^ Parameter $\beta_{VOC}$ was determined by comparing stripping without and with a condenser (dew point at 12.5°C).

^g^ Parameter $\beta_{VOC}$ was estimated for an assumed equilibration temperature of 20°C (see Supporting Information 3).

In order to decrease the expenditure on abiotic stripping experiments, stripping of all VOCs was also performed simultaneously (Supporting Information 4). Here, initial concentrations of the individual VOCs ethyl acetate, ethanol and acetaldehyde were lowered to around 1 g L^−1^ to reduce mutual interferences. The stripping rates of these compounds in the combined stripping experiment complied with the rates determined in the single stripping tests.

#### 3.2.2 Partition coefficient $K_{VOC,L/G}$

The partitioning of a volatile compound between the liquid and the gas phase is specified by diverse constants [48]. The here used partition coefficient is defined as the mass concentration of VOC in the liquid phase divided by the mass concentration of VOC in the gas phase at equilibrium and infinite dilution. In the considered processes, the phase equilibrium was ensured by intensive mixing and the liquid‐phase concentrations were low so that Equation (3) is always valid.

The partition coefficient is usually measured for water/air systems (see Supporting Information 2), but dissolved substances such as minerals, sugar and other organics can exert a high influence on $K_{VOC,L/G}$ [7, 19, 49−56]. Hence, $K_{VOC,L/G}$ values for a given medium/air system have to be determined experimentally.

The wanted partition coefficients of ethyl acetate, ethanol and acetaldehyde for the used cultivation medium at 32°C were calculated from the stripping rates obtained for the system without a condenser (Table 1) using Equation (16), modified for $\beta_{VOC}$ = 0 due to absent VOC retention: $K_{VOC,L/G}=\;-\left(F_{G,R}/V_{L}\right)/k_{VOC}^{without\;c.}$. The specific gas‐flow rate was identical for all stripping experiments and calculated by combining Equations (7) and (8): $F_{G,R}/V_{L}$ = 58.13 L L^−1^h^−1^.

The partition coefficients of the three studied VOCs differed significantly from each other (Table 1); $K_{AA,L/G}$ is more than twice and $K_{EtOH,L/G}$ more than 26 times higher compared to $K_{EA,L/G}$. These differences originate from their individual vapor pressure, hydrophobicity and solubility in the liquid phase. The $K_{VOC,L/G}$ values in the medium/air system were consistently smaller than the respective $K_{VOC,L/G}$ values in water/air at 32°C (the latter originate from literature data as detailed in Supporting Information 2 and were depicted in Figure 1D). This observation is in line with earlier findings [7, 19] and meets the expectations; dissolved inorganic salts [49−52] and sugars [19, 53] reduce the partition coefficient of VOCs. Detailed studies for ethyl acetate demonstrated that an increasing sugar concentration more and more reduced the solubility of the ester in the liquid phase and thus diminished the partition coefficient [19, 54].

Aprea et al. [55] and Ammari et Schroen [56] showed that the presence of ethanol in the aqueous phase increases $K_{EA,L/G}$; ethanol obviously heightens the ester solubility in water. This observation is important since ethanol may emerge as a by‐product during cultivation. Partitioning of ethyl acetate between the culture medium and headspace gas was therefore studied in a non‐aerated bioreactor at 32°C as follows: 3 g L^‒1^ ester were dissolved in the culture medium, rising amounts of ethanol were added step by step, and the effect of this ethanol on $K_{EA,L/G}$ was followed by repeated analysis of the headspace ester concentration. Increasing ethanol concentrations raised the $K_{EA,L/G}$ value as expected (e.g., 3 g L^‒1^ ethanol increased the $K_{EA,L/G}$ by 0.9%). The obtained data were fitted with a function similar to the Setchinow‐Harned‐Owen equation [67]:

$$\begin{array}{ccc} & & K_{\mathrm{EA},L/G}^{\mathrm{with}\;\mathrm{EtOH}}=K_{\mathrm{EA},L/G}^{\mathrm{with}\mathrm{out}\;\mathrm{EtOH}}\;\cdot \exp\left(a\cdot C_{\mathrm{EtOH},L}\right)\\ & & \mathrm{with}\;a=0.00286{L\;g}^{-1}. \end{array}$$

Finally, it is referred to the fact that aldehydes and ketones are hydrated in aqueous solution which reduces the concentration of the free compound [68]. This effect is also observed for acetaldehyde (CH_3_CHO + H_2_O → CH_3_CH(OH)_2_) where 58% of the aldehyde exists in its hydrated form at 25°C [70]. For this reason, it is differentiated between the intrinsic and apparent or effective partition coefficient [48, 50, 68, 70]; the former is the liquid concentration of the free compound divided by the gas‐phase concentration while the latter is the total liquid concentration (sum of the hydrated and non‐hydrated aldehyde) related to the gas‐phase concentration. The here‐used $K_{AA,L/G}$ value is an effective partition coefficient.

#### 3.2.3 Retention efficiency of the condenser $\beta_{VOC}$

A condenser is typically used in aerated bioreactors to avoid excessive loss of water by evaporation. The moist exhaust gas from the bioreactor is cooled when passing the condenser, gaseous water vapor partially condenses and runs back to the reactor. The condensate film in the condenser absorbs some VOCs from the exhaust gas and transports the dissolved VOCs back to the reactor. This transport results in a slowed down VOC stripping and thus decreases the stripping rate. This VOC retention was taken into account by introducing parameter $\beta_{VOC}$ into Equation (1). Calculation of $R_{VOC}\left(t\right)$ and $m_{VOC}\left(t\right)$ from the measured $C_{VOC,G}$ data by Equations (10) and (13) requires $\beta_{VOC}$.

$\beta_{VOC}$ is compound‐specific and depends on the condensation process in the cooler as well. Modeling the VOC retention in a condenser has shown that $\beta_{VOC}$ is mainly determined by $K_{VOC,L/G}$ which, in turn, highly depends on the equilibrium temperature within the condenser (for details see Supporting Information 3). Former stripping experiments for ethyl acetate revealed that retention of the ester was very small and thus unquantifiable resulting in $\beta_{EA}$ ≈ 0 [19], while retention of ethanol was significant [33, 34].

Comparative stripping experiments with and without a condenser allowed the calculation of the retention efficiency for ethanol and acetaldehyde. Equation (16) was specified for the two processes: $\beta_{VOC}$ was kept at stripping with a condenser, and $\beta_{VOC}$ was set to zero at stripping without a condenser. Combining the two modified equations yields after rearrangement: $\beta_{VOC}=\;1-\;k_{VOC}^{with\;c.}/k_{VOC}^{without\;c.}$. In case of a dew‐point temperature of 12.5°C in the condenser, the retention efficiencies were $\beta_{EtOH}$ = 0.147 ± 0.003 and $\beta_{AA}$ = 0.0146 ± 0.0003.

The experimentally determined values of $\beta_{VOC}$ correlate well with the values which were estimated by a model derived in Supporting Information 3 (Table 1). The VOC retention is mainly determined by its partition coefficient (Equation (S3.16) in Supporting Information 3). Therefore, retention of ethyl acetate is negligible, retention of acetaldehyde is noticeable but small (Figure 1B), while retention of ethanol is significant (Figure 1C).

### 3.3 Synthesis of ethyl acetate by *K. marxianus* DSM 5422 as an example

Balancing the synthesis/degradation of VOCs is explained in detail in Section 3.1 and is here applied to the aerobic batch cultivation of *K. marxianus* DSM 5422 in a stirred and aerated 1‐L bioreactor. This yeast strain has been proven as a potent producer of ethyl acetate from whey‐borne sugars [7, 19−25]. Here, a glucose‐based mineral medium supplemented with vitamins was used since growth of *K. marxianus* DSM 5422 in whey‐based DW medium became limited by an unknown factor, most likely by a missing vitamin [61]. The growth of *K. marxianus* and the ester synthesis was studied under iron‐limited conditions (ester synthesis by *K. marxianus* DSM 5422 is induced by a lack of iron [21]) and under non‐limited conditions as a reference. Dissolved oxygen (DO) was continuously measured for monitoring the oxygenation state of the culture. The medium was intensively stirred in both experiments so that the DO value was always above 40% air saturation.

#### 3.3.1 Growth behavior depending on available iron

**FIGURE 2:**
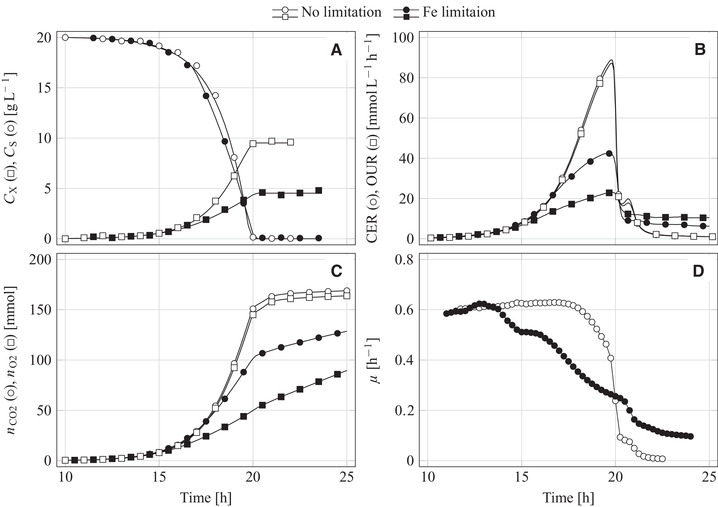
(A) Sugar and biomass concentration, (B) O_2_ uptake rate (OUR) and CO_2_ evolution rate (CER), (C) cumulative O_2_ consumption and CO_2_ formation, and (D) the specific growth rate derived from O_2_‐consumption data during the aerobic batch cultivation of *K. marxianus* DSM 5422 in a 1‐L stirred bioreactor using 0.6 L glucose‐based mineral medium; Cultivation under non‐limited conditions (medium with FeSO_4_) or under iron‐limited conditions (medium without FeSO_4_)

The reference experiment was characterized by exponential yeast growth until glucose became a limiting factor (Figure 2A). In the iron‐limited experiment, the growth started exponentially as well but later changed into a linear growth mode (Figure 2A). Iron limitation reduced the final cell concentration compared to the reference experiment (4.5 vs. 9.5 g L^−1^) but the courses of sugar consumption were nearly the same in both processes (Figure 2A).

Significant yeast growth despite omission of iron from the medium is explained by iron that originates from media constituents (as already observed by Thomas et Dawson [44]). Shake‐flask experiments demonstrated that the iron impurities of the media constituents allow the observed yeast growth (not shown). Moreover, dissolution of some iron from the stainless steel of the bioreactor internals can not be ruled out; such a dissolution was detected during cultivation of *K. marxianus* DSM 5422 at an elevated temperature of 42°C [23].

Continuously measured O_2_ and CO_2_ concentrations in the exhaust gas were used to calculate O_2_ consumption and CO_2_ formation rates according to Duboc et von Stockar [33]. These rates are denoted as the O_2_ uptake rate (OUR(t)) and the CO_2_ evolution rate (CER(t)) and are given in moles of consumed O_2_ or formed CO_2_ per liter reaction volume and hour. The CER(t)‐OUR(t) ratio represents a momentary respiratory quotient, RQ(t). The reference process was completely respiratory as seen from the nearly identical OUR(t) and CER(t) courses, yielding RQ ≈ 1 mol mol^−1^ (Figure 2B). The iron‐limited process also started respiratory but later shifted to a partially fermentative sugar utilization; CER(t) increased quicker than OUR(t), and the RQ value became >1 mol mol^−1^. After a process time of 20 h, glucose was depleted in both processes which resulted in sharp decreases of OUR(t) and CER(t). Some O_2_ consumption and CO_2_ formation after glucose depletion was connected with utilization of dissolved metabolites.

Momentary values of OUR(t) and CER(t) multiplied by the liquid volume and integrated over time yielded cumulative amounts of consumed O_2_ and formed CO_2_, $n_{O_{2}}\left(t\right)$ and $n_{CO_{2}}\left(t\right)$, respectively. The $n_{O_{2}}\left(t\right)$ and $n_{CO_{2}}\left(t\right)$ courses were similar in the reference experiment, but distinctly differed from each other in the iron‐limited experiment reasoned by the partial fermentation beginning at a process time of around 14 h (Figure 2C).

Time‐dependent specific growth rates, μ(t), were calculated from the $OD_{600nm}\left(t\right)$, $n_{O_{2}}\left(t\right)$ and $n_{CO_{2}}\left(t\right)$ data as described in [61]. Determination of μ(t) from $OD_{600nm}$ data requires sufficiently high cell densities, while μ(t) calculations from $n_{O_{2}}\left(t\right)$ and $n_{CO_{2}}\left(t\right)$ work also well in an early growth stage at low cell densities. However, the determination of μ(t) from $n_{CO_{2}}\left(t\right)$ is only permissible at respiratory sugar utilization since additional CO_2_ formation at a partially fermentative process results in over‐estimated μ values, while μ(t) acquisition from the O_2_ data are less susceptible in this respect. After sugar depletion, determination of μ(t) from $n_{O_{2}}\left(t\right)$ is falsified due to microbial oxidation of dissolved metabolites.

Both processes started with exponential growth as shown by the μ(t) courses (Figure 2D). Maximum growth rates ($\mu_{max}$) calculated for this exponential period were nearly independent of the used data basis ($n_{CO_{2}}\left(t\right)$, $n_{O_{2}}\left(t\right)$ or OD(t)), and were identical for both processes. In the reference experiment, $\mu_{max}$ amounted to 0.614, 0.615 and 0.600 h^−1^, and in the iron‐limited experiment $\mu_{max}$ was 0.615, 0.621 and 0.612 h^−1^. These $\mu_{max}$ values are well comparable with the maximum specific growth rate of *K. marxianus* DSM 5422 observed in highly diluted whey‐based DW medium at 32°C [22, 23, 72].

In the reference experiment, yeast growth occurred with $\mu_{max}$ until glucose became a growth‐limiting factor while in the other experiment, a deficit of iron reduced the growth rate long before sugar depletion (Figure 2D) as already observed earlier [19]. Iron‐limited yeast growth depends directly on the intracellular iron content and only indirectly on the extracellular iron concentration. Growth depending on intracellular iron and iron uptake are two decoupled processes [61]. A limited available essential trace metal is taken up quickly at the onset of cultivation and stored in the cell; continued growth after depletion of the trace metal in the culture medium is then based on the stored metal, accompanied by a steady reduction of the intracellular metal content. This mechanism was studied for zinc during fermentation of *Saccharomyces* strains in malt wort [73, 74] and is also valid for iron‐dependent growth of *K. marxianus* DSM 5422 [61]. The period of iron‐limited glucose utilization lasted here approximately 6 h (Figures 2A,D).

#### 3.3.2 Synthesis of volatile metabolites

Iron limitation as an inductor for the synthesis of ethyl acetate in *K. marxianus* and other yeasts has been known for a long time [7, 8, 14, 17−24, 27, 44, 75]. Synthesis of ethyl acetate and other VOCs were here detected by regular gas‐chromatographic analysis of the exhaust gas of the bioreactor, yielding $C_{VOC,G}\left(t\right)$ data.

**FIGURE 3:**
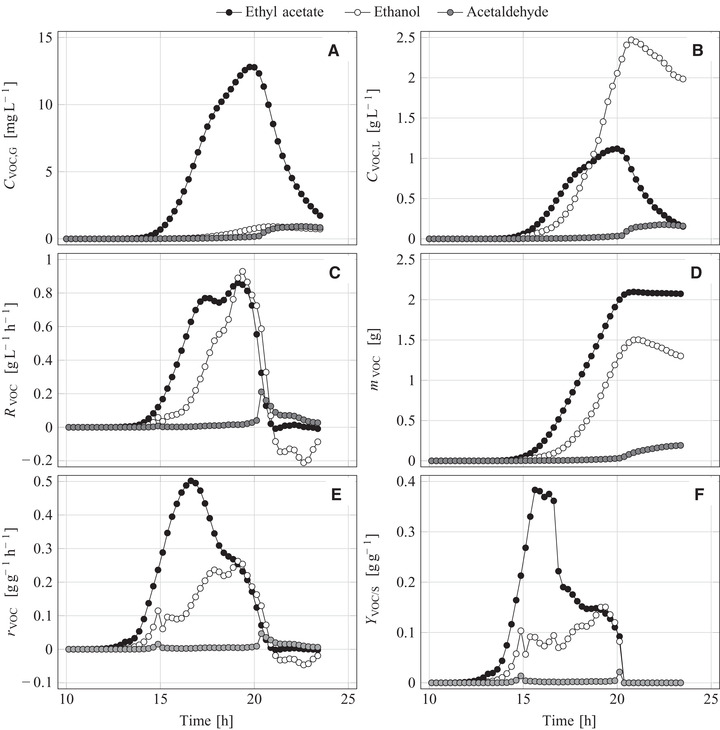
Concentrations of volatile metabolites (A) in the exhaust gas as measured and (B) in the liquid phase, (C) volume‐specific reaction rates, (D) cumulative masses of formed volatile metabolites, (E) biomass‐specific reaction rates, and (F) yields of volatile metabolites during the aerobic batch cultivation of *K. marxianus* DSM 5422 in a 1‐L stirred bioreactor using 0.6 L glucose‐based mineral medium under iron‐limited conditions (medium without FeSO_4_)

In the reference experiment, small amounts of ethyl acetate were formed with a basal synthesis rate ($C_{EA,G}$ = 2.1 mg L^−1^ at the most) while formation of ethanol and acetaldehyde were negligible. In the iron‐limitation experiment, synthesis of ethyl acetate started along with the growth‐rate reduction (Figure 3A, maximum $C_{EA,G}$ of 12.8 mg L^−1^). The measured $C_{VOC,G}\left(t\right)$ data were transformed into liquid‐phase concentrations by using Equation (9), assuming a phase equilibrium between both phases because of intensive stirring (Figure 3B). The calculated $C_{VOC,L}$ values were verified by GC analyses of the VOCs in the culture medium (Supporting Information 1, Figure S1.1). The $C_{VOC,L}\left(t\right)$‐$C_{VOC,G}\left(t\right)$ ratio (compare Figure 3B with Figure 3A) highly depends on the volatile compound since this relation is mainly determined by the compound‐specific partition coefficient and a little influenced by the specific VOC retention in the condenser (Table 1, Equation (9)). The $C_{VOC,L}\left(t\right)$‐$C_{VOC,G}\left(t\right)$ ratio could also change during the process due to temporal variation of the partition coefficient, $K_{VOC,L/G}$, because of a changing medium composition reasoned by sugar consumption and NaOH dosage for pH regulation (for more details see Section 3.2.2). Previous cultivation experiments have clearly shown that such $K_{VOC,L/G}$ variation over time is insignificant (Supporting Information 5). The quick drop of the ester concentration in both phases (Figures 3A,B) is reasoned by ester stripping but not by ester degradation (details below).

The measured $C_{VOC,G}\left(t\right)$ data were further transformed into time‐dependent volume‐specific reaction rates ($R_{VOC}\left(t\right)$, using Equation (10)) and masses of cumulatively formed VOCs ($m_{VOC}\left(t\right)$, using Equation (13)). $R_{VOC}$ is an engineering process parameter that provides information about the efficiency of VOC synthesis and is given as the mass of formed VOC per liter culture volume and hour (Figure 3C). Besides the ester synthesis (2.13 g vs. 0.28 g in the reference experiment), the iron limitation also induced ethanol formation (1.52 g vs. 0.04 g), but the ethanol synthesis started with a delay of 1.5 h (Figures 3C,D). Under normal circumstances, *K. marxianus* as a Crabtree‐negative yeast does not produce ethanol under aerobic conditions [76−79] with the exception of a deregulated cell metabolism [80] as it is the case under iron limitation. Simultaneous synthesis of ethanol and ethyl acetate was frequently observed in previous studies despite fully aerobic conditions [8, 17, 19−24].

Formed ethanol was metabolized after depletion of sugar ($R_{EtOH}\left(t\right)$ became negative and $m_{EtOH}\left(t\right)$ declined at t >20 h) while ethyl acetate was not microbially consumed at all ($R_{EA}\left(t\right)$ always ≥0). Larger amounts of acetaldehyde were only produced after sugar depletion, obviously via microbial oxidation of ethanol (Figures 3C,D). Synthesis of acetaldehyde by *K. marxianus* in iron‐deficient whey‐based medium was also observed by Kallel‐Mhiri et al. [17].

The $R_{VOC}\left(t\right)$ data were related to the biomass concentration ($C_{X}\left(t\right)$ data in Figure 2A) yielding biomass‐specific reaction rates ($r_{VOC}\left(t\right)$, using Equation (11)). These rates are biological parameters denoting for the masses of formed/consumed VOCs per gram of biomass and hour. The intensity of ester synthesis is best evaluated via $r_{EA}\left(t\right)$. The onset of iron limitation induced a steep rise in $r_{EA}\left(t\right)$ which reached a maximum at t = 16.7 h under moderate iron limitation, and then continuously decreased (Figure 3E). The maximum $r_{EA}\left(t\right)$ value amounted to 502 mg g^−1^h^−1^ which is in line with earlier findings for *K. marxianus* DSM 5422 in whey‐based medium [21, 24]. The synthesis rate in the reference experiment was much lower (maximum $r_{EA}\left(t\right)$ = 38 mg g^−1^h^−1^). Synthesized ethyl acetate was not stripped instantly but temporarily accumulated in the culture medium (Figure 3B) and, thus, could have been degraded by the yeast. However, the $r_{EA}\left(t\right)$ course (Figure 3E) ruled out significant degradation (minimum $r_{EA}$ = ‒2 mg g^−1^h^−1^). In contrast to $r_{EA}\left(t\right)$, $r_{EtOH}\left(t\right)$ increased slower, reached a maximum short before sugar depletion, and then became negative due to degradation of ethanol.

$R_{VOC}\left(t\right)$ was used to calculate time‐dependent yields of the volatiles ($Y_{VOC/S}\left(t\right)$ calculated by Equation (14)). The $Y_{VOC/S}\left(t\right)$ courses distinctly varied over time (Figure 3F). $Y_{EA/S}\left(t\right)$ at first increased, reached a maximum at t = 15.5 to 16.5 h, and then decreased until sugar depletion. The detected maximum yield of *K. marxianus* DSM 5422 amounted to 0.38 g g^−1^ (Figure 3F), which corresponds to 78% of the theoretical maximum. The overall yield of ethyl acetate as an average value was, however, not really high (0.182 g g^−1^) due to non‐optimized process conditions.

## 4 CONCLUDING REMARKS

In this study, an innovative method was developed for model‐based balancing of volatile metabolites. Its functionality was demonstrated for ethyl acetate, ethanol, and acetaldehyde synthesized during iron‐limited batch cultivation of *K. marxianus* DSM 5422 in an aerated bioreactor. This new approach is solely based on the measurement of VOC concentrations in the exhaust gas and allows the calculation of formed masses, reaction rates and yields of VOCs in a high temporal resolution.

The $K_{VOC,L/G}$ and $\beta_{VOC}$ parameters required for these calculations were determined separately for each VOC by two stripping experiments using a bioreactor with and without condenser. Actually, only the $K_{VOC,L/G}/\left(1-\beta_{VOC}\right)$ term must be known for each VOC since this term is an element of all calculation equations, while $K_{VOC,L/G}$ and $\beta_{VOC}$ do not appear separated. The needed term is determinable by a stripping experiment using the bioreactor with condenser. The wanted term is obtained from the measured stripping rate and the applied specific gas‐flow rate (according to Equation (16)): $K_{VOC,L/G}/\left(1-\beta_{VOC}\right)$ = $-\left(F_{G,R}/V_{L}\right)/k_{VOC}$. It was also demonstrated that the experimental effort can be even more reduced when the stripping of all VOCs of interest is studied in a single experiment. In this experiment, the initial VOC concentrations was low (about 1 g L^‒1^ ethyl acetate, ethanol and acetaldehyde in the liquid) to avoid interferences between the volatiles. The stripping rates obtained from single and combined stripping experiments were identical (Supporting Information 5).

The temporal resolution of VOC measurements amounted to 15‐min intervals. This resolution could be even improved when VOCs are analyzed together with O_2_ and CO_2_ via a mass spectrometer. Quantification of stripped ethyl acetate by mass spectrometry has already been demonstrated by Bohnenkamp et al. [31].

**Table:** 

**Nomenclature**		
**Symbol**	**Unit**	**Description**
$C_{EA,G}$	[g L^−1^]	Concentration of ethyl acetate in the gas at the gas‐line exit
$C_{EA,G,R}$	[g L^−1^]	Concentration of ethyl acetate in the headspace gas
$C_{EA,L}$	[g L^−1^]	Concentration of ethyl acetate in the cultivation medium
$C_{EtOH,L}$	[g L^−1^]	Concentration of ethanol in the cultivation medium
$C_{S}$	[g L^−1^]	Sugar concentration in the cultivation medium
$C_{VOC,G}$	[g L^−1^]	Concentration of a VOC in the gas at the gas‐line exit
$C_{VOC,G,R}$	[g L^−1^]	Concentration of a VOC in the headspace gas
$C_{VOC,L}$	[g L^−1^]	Concentration of a VOC dissolved in the cultivation medium
$C_{X}$	[g L^−1^]	Biomass concentration in the cultivation medium given as dry weight
CER	[mol L^−1^h^−1^]	Carbon dioxide evolution rate
DO	[%]	Dissolved‐oxygen concentration related to the saturation concentration
$F_{G}$	[L h^−1^]	Gas flow leaving the bioreactor system at the gas‐line exit
$F_{G,0}$	[L h^−1^]	Gas flow supplied to the bioreactor (given for 273.15 K and 101325 Pa)
$F_{G,R}$	[L h^−1^]	Gas flow leaving the bioreactor at headspace conditions
$K_{AA,L/G}$	[L L^−1^]	Partition coefficient of acetaldehyde in the bioreactor
$K_{EA,L/G}$	[L L^−1^]	Partition coefficient of ethyl acetate in the bioreactor
$K_{EtOH,L/G}$	[L L^−1^]	Partition coefficient of ethanol in the bioreactor
$K_{O/W}$	[L L^−1^]	Partition coefficient of a VOC between n‐octanol and water
$K_{VOC,L/G}$	[L L^−1^]	Partition coefficient of a VOC between liquid and gas in the bioreactor
$k_{AA}$	[h^−1^]	Stripping rate of acetaldehyde from the bioreactor
$k_{EA}$	[h^−1^]	Stripping rate of ethyl acetate from the bioreactor
$k_{EtOH}$	[h^−1^]	Stripping rate of ethanol from the bioreactor
$k_{VOC}$	[h^−1^]	Stripping rate of a VOC from the bioreactor
$k_{VOC}^{with\;c.}$	[h^−1^]	Stripping rate of a VOC from the bioreactor fitted with condenser
$k_{VOC}^{without\;c.}$	[h^−1^]	Stripping rate of a VOC from the bioreactor without condenser
$k_{VOC,L}a$	[h^−1^]	Phase‐transfer coefficient of a VOC in the bioreactor
$m_{EA}$	[g]	Mass of cumulatively synthesized/degraded ethyl acetate
$m_{VOC}$	[g]	Mass of cumulatively synthesized/degraded VOC
$n_{CO_{2}}$	[mol]	Moles of cumulatively formed carbon dioxide
$n_{O_{2}}$	[mol]	Moles of cumulatively consumed oxygen
OUR	[mol L^−1^h^−1^]	Oxygen uptake rate
$R_{EA}$	[g L^−1^h^−1^]	Volume‐specific rate of ethyl acetate synthesis/degradation
$R_{S}$	[g L^−1^h^−1^]	Volume‐specific rate of sugar consumption
$R_{VOC}$	[g L^−1^h^−1^]	Volume‐specific rate of VOC synthesis/degradation
RQ	[mol mol^−1^]	Respiratory quotient
$r_{AA}$	[g g^−1^h^−1^]	Biomass‐specific rate of acetaldehyde synthesis/degradation
$r_{EA}$	[g g^−1^h^−1^]	Biomass‐specific rate of ethyl acetate synthesis/degradation
$r_{EtOH}$	[g g^−1^h^−1^]	Biomass‐specific rate of ethanol synthesis/degradation
$r_{VOC}$	[g g^−1^h^−1^]	Biomass‐specific rate of VOC synthesis/degradation
$T_{G}$	[K]	Temperature of gas at the gas‐line exit
$T_{G,0}$	[K]	Temperature of the supplied gas flow (273.15 K)
$T_{G,R}$	[K]	Temperature of the headspace gas
t	[h]	Process time
$V_{L}$	[L]	Volume of the cultivation medium in the bioreactor
$x_{CO_{2},G}$	[L L^−1^]	CO_2_ content in the gas at the gas‐line exit
$x_{CO_{2},G,0}$	[L L^−1^]	CO_2_ content in the supplied gas flow
$x_{O_{2},G}$	[L L^−1^]	O_2_ content in the gas at the gas‐line exit
$x_{O_{2},G,0}$	[L L^−1^]	O_2_ content in the supplied gas flow
$x_{W,G}$	[L L^−1^]	Water content in the gas at the gas‐line exit
$x_{W,G,0}$	[L L^−1^]	Water content in the supplied gas flow
$x_{W,G,R}$	[L L^−1^]	Water content in the headspace gas
$Y_{EA/S}$	[g g^−1^]	Yield of ethyl acetate
$Y_{EA/S,max}$	[g g^−1^]	Maximum yield of ethyl acetate
$Y_{EtOH/S}$	[g g^−1^]	Yield of ethanol
$Y_{VOC/S}$	[g g^−1^]	Yield of a VOC
**Greek symbols**		
$\beta_{AA}$	−	Relative retention of acetaldehyde by the exhaust‐gas condenser
$\beta_{EA}$	−	Relative retention of ethyl acetate by the exhaust‐gas condenser
$\beta_{EtOH}$	−	Relative retention of ethanol by the exhaust‐gas condenser
$\beta_{VOC}$	−	Relative retention of a VOC by the exhaust‐gas condenser
Δt	[h]	Time interval
μ	[h^−1^]	Specific growth rate
$\mu_{max}$	[h^−1^]	Maximum specific growth rate
τ	[h]	Control variable at integration

## CONFLICT OF INTEREST

The authors have declared no conflict of interest.

## Supporting information



Supporting Information

## Data Availability

The data that support the findings of this study are available from the corresponding author upon reasonable request.
