# Supplementary material for: A new approach for balancing the microbial synthesis of ethyl acetate and other volatile metabolites during aerobic bioreactor cultivations
Source: Eng Life Sci. 2020 Dec 21;21(3-4):137–53. doi: 10.1002/elsc.202000047 (PMC7923609; doi:10.1002/elsc.202000047)
Supplement: Supplementary file 1 — Supporting Information [file ELSC-21-137-s001.pdf]

Supporting Information 1: $C_{VOC,L}$-$C_{VOC,G}$ ratio

A new approach for balancing the microbial synthesis of ethyl acetate and other volatile metabolites during aerobic bioreactor cultivations

Christian Löser, Christian Kupsch, Thomas Walther, Andreas Hoffmann

Used symbols are listed in the main part of the work or are explained here in the text.

The quantitative evaluation of VOC synthesis requires determination of time-dependent yields and biomass-specific reaction rates (synthesis or consumption rates). The calculation of these variables was formerly based on the concentrations of the considered VOCs in the exhaust gas, $C_{VOC,G}$, as well as in the liquid phase, $C_{VOC,L}$, both depending on time [Urit et al. 2011; Urit et al. 2012; Löser et al. 2012; Löser et al. 2014]. Determination of the wanted yields and reaction rates in a high temporal resolution requires repeated measurement of both VOC concentrations at short intervals. Such measurements are very time and labor-consuming. Especially the liquid-sample analyses by gas-chromatography are elaborate and inaccurate. Then again, non-accurate measurements cause a high fluctuation of calculated rates.

For this reason, the liquid-phase concentrations were not measured but calculated from the analyzed gas-phase concentrations based on the fact that both concentrations are in a fixed ratio at consistent cultivation conditions (provided that the system is nearly equilibrated). The gas-phase concentrations are quickly to measure by gas-chromatography within a period of less than 5 min, they are more precise than liquid-phase GC measurements, and gas sampling at the outlet of the bioreactor does not influence the process.

Next, it is explained how the liquid-phase concentration of a VOC is calculated from the measured gas-phase concentration. The residence time of gas that flows from the headspace of bioreactor through the condenser to the sampling port is negligible. A measured gas-phase concentration can thus be converted into a respective headspace concentration which in turn is transformed into a liquid-phase concentration, assuming a phase equilibrium between the headspace gas and culture medium within the bioreactor (which verifiably exists due to intensive mixing of both phases).

According to Equation (3) in the main text, there exists a constant relation between the liquid-phase and gas-phase concentration in the bioreactor which allows calculation of liquid-phase concentrations:

$$C_{VOC,L} = K_{VOC,L/G} \cdot C_{VOC,G,R} \qquad \text{at intensive mixing} \qquad \text{(S1.1)}$$

Equation (5) in the main text describes the relation between the headspace-gas concentration and the gas concentration of the VOC at the sampling port behind the condenser (changes of the gas flow and a partial VOC retention by the condenser take influence on the gas-phase concentration):

$$C_{VOC,G,R} = C_{VOC,G} \cdot \frac{F_G}{F_{G,R}} \cdot \frac{1}{1-\beta_{VOC}} \qquad \text{(S1.2)}$$

Combination of equations (S1.1) and (S1.2) yields:

$$C_{VOC,L} = C_{VOC,G} \cdot \frac{F_G}{F_{G,R}} \cdot \frac{K_{VOC,L/G}}{1-\beta_{VOC}} \qquad \text{(S1.3)}$$

According to Equation (7) in the main text, there exists a constant relation between the two gas flows F_G and $F_{G,R}$, considering the thermal effect and partial dehumidification in the condenser:

$$\frac{F_G}{F_{G,R}} = \frac{T_G}{T_{G,R}} \cdot \frac{1-x_{W,G,R}}{1-x_{W,G}} \qquad \text{(S1.4)}$$

Combining Equations (S1.3) and (S1.4) results in the wanted relation:

$$C_{VOC,L} = C_{VOC,G} \cdot \frac{T_G}{T_{G,R}} \cdot \frac{1 - x_{W,G,R}}{1 - x_{W,G}} \cdot \frac{K_{VOC,L/G}}{1 - \beta_{VOC}} \quad \text{(S1.5)}$$

All parameters in this equation do not change during the cultivation process so that a constant relation between both concentrations exists. The bioreactor experiments described in the main text were performed under the following conditions:

T_G = 298.15 K

$T_{G,R}$ = 305.15 K

$x_{W,G,R}$ = 0.0469 L L^{-1}

$x_{W,G}$ = 0.0143 L L^{-1}

The $K_{VOC,L/G}$ parameter depends on the species of VOC, on the reaction temperature and on the nature of the liquid phase; $K_{VOC,L/G}$ values have been determined experimentally by stripping experiments for ethyl acetate, ethanol and acetaldehyde at $T_{G,R}$ = 32 °C for the medium/air system and are given in Table 1 of the main text. This table also contains experimentally determined β_{VOC} values for the three named VOCs (VOC retention in the condenser with a dew point for water of 12.5 °C). These data result in the $C_{VOC,L}$-$C_{VOC,G}$ ratios listed in the following table:

Table S1.1. Calculated $C_{VOC,L}$-$C_{VOC,G}$ ratios for relevant volatile organic compounds using the above-given parameters and data from Table 1 in the main text

Compound	$K_{VOC,L/G}$ [L L^{-1}]	β_{VOC} [–]	$C_{VOC,L}$-$C_{VOC,G}$ ratio [L L^{-1}]
Ethyl acetate	92.3	0.0000	87.2
Acetaldehyde	194.5	0.0147	186.5
Ethanol	2430	0.1466	2690

The calculated liquid-phase concentrations were finally compared with real liquid-phase VOC concentrations measured by GC analysis as explained in the main text. This comparison was exemplarily done for a process of batch cultivation of *K. marxianus* DSM 5422 in a 1-L stirred bioreactor using 0.6 L glucose-based mineral medium under iron-limited conditions (medium without $FeSO_4$ supplementation). This process is depicted in Figs. 2 and 3 of the main text. Regarding the analysis and calculation procedures it is also referred to the main text.

It becomes clearly visible from Fig. S1.1 that the calculated liquid-phase VOC concentrations correspond very well to the really measured ethanol, ethyl acetate and acetaldehyde concentrations. Some deviations are explained by measuring errors. To our experience, the GC analysis of liquid samples are distinctly more error-prone that the GC analysis of gas samples, being mainly explained by the smaller analysis volume, the required pre-dilution of sugar-rich and thus viscous samples, and the mixing with an internal standard before analysis.

The calculated dissolved VOC concentrations match the measured values, therefore, liquid-phase concentrations of VOCs can be calculated as described in the main text instead of being measured. This validates the proposed model-based method for balancing VOCs only by means of measured gas-phase concentrations.

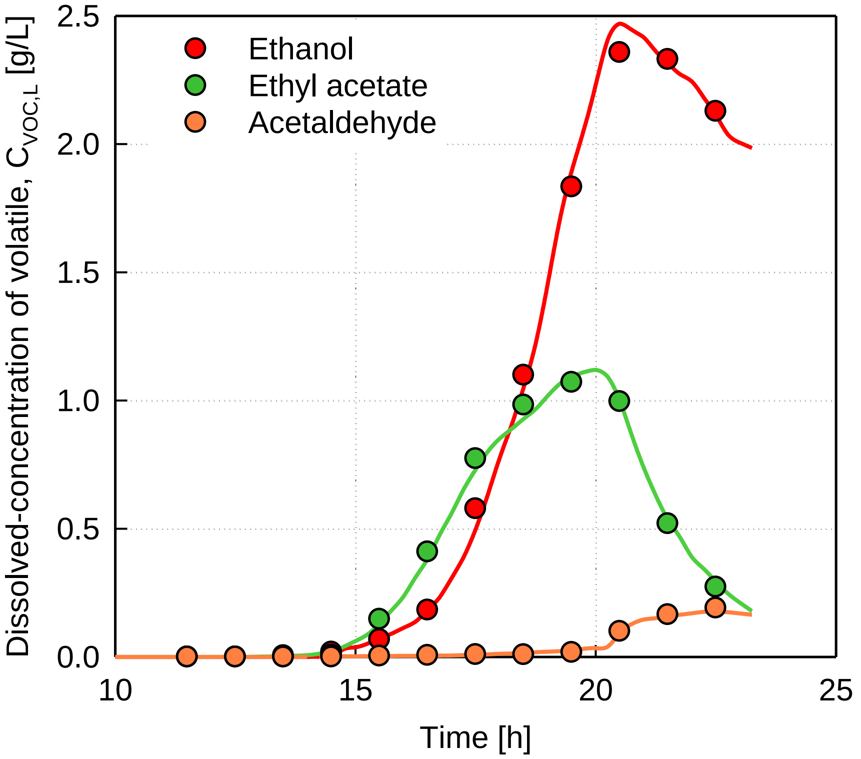

Figure S1.1. Measured concentrations of the volatile metabolites ethanol, ethyl acetate and acetaldehyde in the liquid phase (symbols) together with the liquid-phase concentrations of these volatiles calculated from their respective measured gas-phase concentrations (continuous lines) during the aerobic batch cultivation of *K. marxianus* DSM 5422 in a 1-L stirred bioreactor using 0.6 L glucose-based mineral medium under iron-limited conditions (medium without $FeSO_4$).

References

Löser, C., Urit, T., Förster, S., Stukert, A., Bley, T., Formation of ethyl acetate by *Kluyveromyces marxianus* on whey during aerobic batch and chemostat cultivation at iron limitation. *Appl. Microbiol. Biotechnol.* 2012, *96*, 685–696.

Löser, C., Urit, T., Bley, T., Perspectives for the biotechnological production of ethyl acetate by yeasts. *Appl. Microbiol. Biotechnol.* 2014, *98*, 5397–5415.

Urit, T., Löser, C., Wunderlich, M., Bley, T., Formation of ethyl acetate by *Kluyveromyces marxianus* on whey: studies of the ester stripping. *Bioprocess Biosyst. Eng.* 2011, *34*, 547–559.

Urit, T., Stukert, A., Bley, T., Löser, C., Formation of ethyl acetate by *Kluyveromyces marxianus* on whey during aerobic batch cultivation at specific trace-element limitation. *Appl. Microbiol. Biotechnol.* 2012, *96*, 1313–1323.

Supporting Information 2: Published partition coefficients

A new approach for balancing the microbial synthesis of ethyl acetate and other volatile metabolites during aerobic bioreactor cultivations

Christian Löser, Christian Kupsch, Thomas Walther, Andreas Hoffmann

Used symbols are listed in the main part of the work or are explained here in the text.

Conditions

Partition coefficients are compound-specific and highly depend on the temperature and the composition of the liquid phase. Here, partitioning data are summarized from the literature with following restrictions:

Nature of data: Restriction to original measurements

Phase system: Water/air system (air-saturated water equilibrated with water-saturated air)

Compounds: Ethyl acetate, ethanol, acetaldehyde

Unit: All data from the literature were transformed into the so-called dimensionless Henry solubility which, strictly speaking, possesses the unit L of gas per L of liquid (here liter air per liter water). This partition coefficient is the ratio between the aqueous-phase concentration and the gas-phase concentration (volume-related masses or moles of the considered compound):

$$K_{i,L/G}(T) = \frac{C_{i,L}}{C_{i,G}} \qquad \text{(S2.1)}$$

Data conversion: Data conversion assumed that the gaseous compounds behaved like an ideal gas. The temperature dependency of the densities of air and water was taken into account as well.

Temperature range: Here, only data in the temperature range from 0 to 40 °C were used.

Solute concentration: All VOC solutions are considered to be highly diluted so that the density of the aqueous VOC solution is nearly identical with the density of pure water.

Effective coefficients: The given partition coefficients are not intrinsic but effective coefficients. This means that the given liquid-phase concentrations are always the sum of both hydrated and non-hydrated species of the considered compound related to the liquid volume. Such hydration occurs with ketones and aldehydes (for details see [Ji et Evans 2007; Sander 2015]).

Equations for empirical data fits

Measured partition data are often fitted by empirical temperature-dependent equations for receiving continuous functions which allow calculation of a partition coefficient for a specific temperature within a given range. The most commonly used function for describing the temperature dependency of the Henry's law constant is based on the van´t Hoff equation in the following form [Sander 2015]:

$$H(T) = A \cdot exp\left(\frac{B}{T}\right) \qquad \text{(S2.2)}$$

Herein, A and B are compound-specific constants. When the Henry constant is transformed into a partition coefficient as defined above then another temperature dependency comes along since the gas density highly depending on temperature which modifies the function (for more details see [Sander 2015]):

$$K_{VOC,L/G}(T) = A^* \cdot T \cdot exp\left(\frac{B}{T}\right) \tag{S2.3}$$

This conversion also modifies the compound-specific constant A into the A^* parameter, but B does not change.

Another enhanced function was recently published by Fenclová et al. [2014] for describing the temperature-dependent Henry's law volatility of esters given in kPa (which is comparable to Eq. No. 101 of Brockbank [2013]):

$$K_H(T) = exp\left(A_H + \frac{B_H}{\tau} + C_H \cdot ln(\tau)\right) \quad \text{with} \quad \tau = \frac{T}{298.15\,K} \tag{S2.4}$$

Variable τ represents the absolute temperature T divided by a standard temperature (here 298.15 K), and A_H, B_H and C_H are compound-specific constants. Transformation of this function results in an equation that describes the temperature dependency of the partition coefficient:

$$K_{VOC,L/G}(T) = a \cdot exp\left(\frac{b}{T}\right) \cdot \left(\frac{T}{T_0}\right)^c \quad \text{with} \quad T_0 = 273.15\,K \tag{S2.5}$$

Herein, a, b and c are compound-specific constants, T is the temperature given in K, and T_0 is the temperature at standard conditions. Equation (S2.5) differs from Equation (S2.3) in so far that the linear temperature factor is replaced by a T^c expression.

Partition coefficients from the literature and data fits

Measured partitioning data from the literature for water/air systems in the temperature range from 0 to 40 °C were transformed into $K_{VOC,L/G}$ values. Such data for ethyl acetate, ethanol and acetaldehyde are depicted in the Figures S2.1 to S2.3. The data were then fitted for each compound by using the $K_{VOC,L/G}(T)$ function given in Equation (S2.5). The obtained parameters a, b and c are listed in Table S2.1 together with the R^2 values.

The scattering of the published data are different for the three considered compounds which is also exhibited by the R^2 values in Table S2.1. The data fit for ethanol is most reliable followed by the fit for ethyl acetate, while the quite low R^2 value of acetaldehyde corresponds to the significant variation of the partition data in Figure S2.3.

Table S2.1. Compound-specific constants of Equation (S2.5) for describing the temperature dependency of the partition coefficients of ethyl acetate, ethanol and acetaldehyde in the water/air system being valid for a temperature range from 273.15 to 313.15 K (0 to 40 °C); R^2 is the coefficient of determination.

Compound	a [L L^{-1}]	b [K]	c [–]	R^2 [–]
Ethyl acetate	$4.007 \cdot 10^{-22}$	15302.9	33.94	0.9917
Ethanol	$1.912 \cdot 10^{-16}$	12718.8	22.32	0.9984
Acetaldehyde	$1.913 \cdot 10^{-6}$	5651.4	1	0.9563

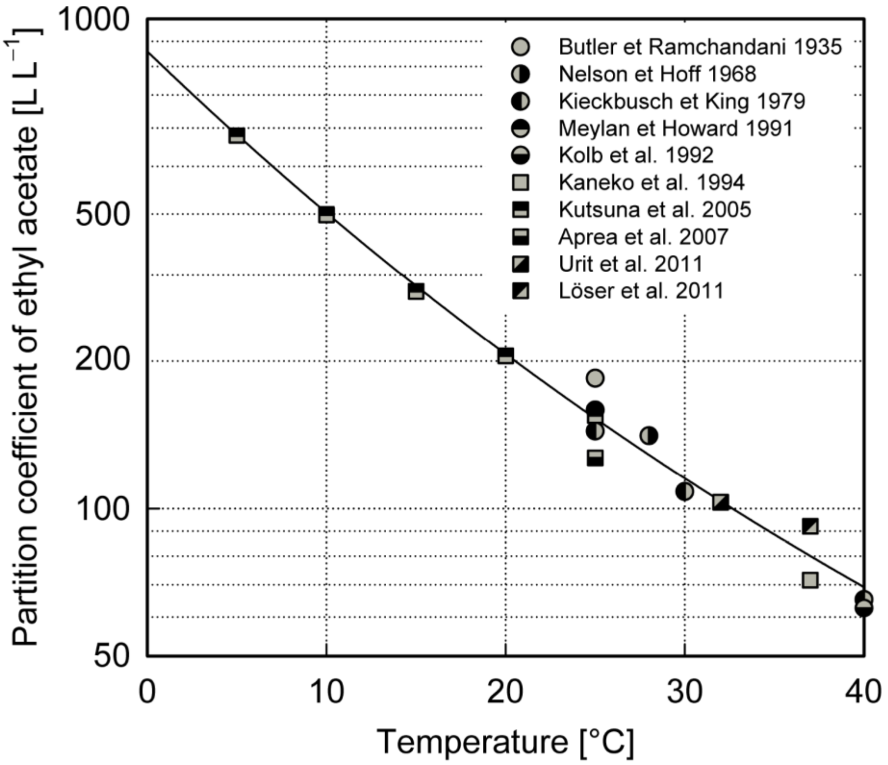

Figure S2.1. Partition coefficients of ethyl acetate in the water/air system taken from the literature and fitted with Equation (S2.5) resulting in the compound-specific constants given in Table S2.1.

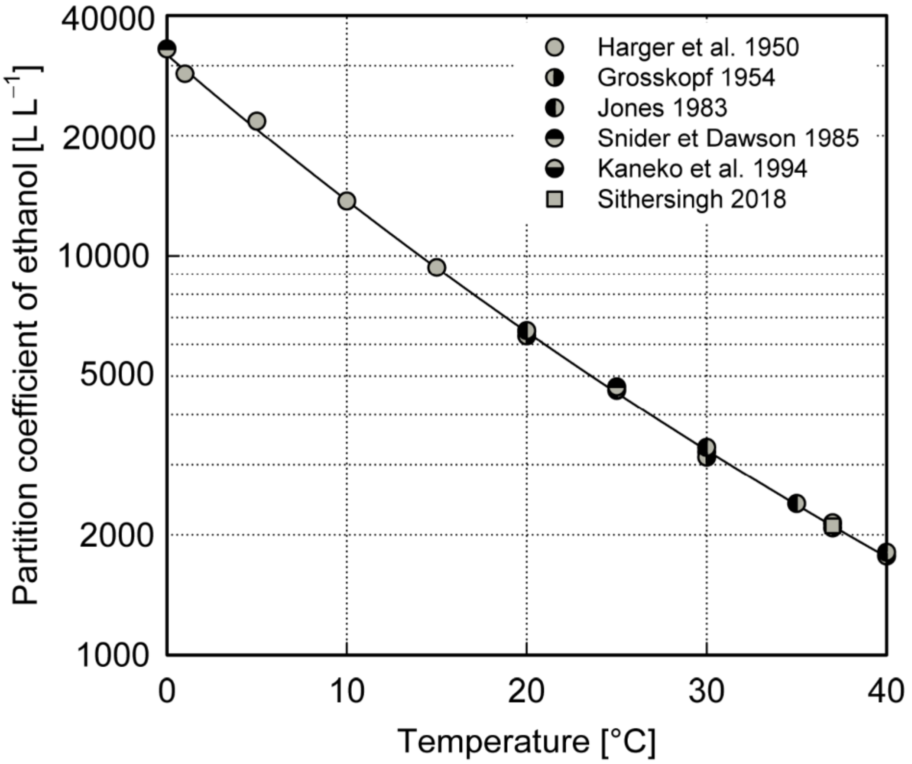

Figure S2.2. Partition coefficients of ethanol in the water/air system taken from the literature and fitted with Equation (S2.5) resulting in the compound-specific constants given in Table S2.1.

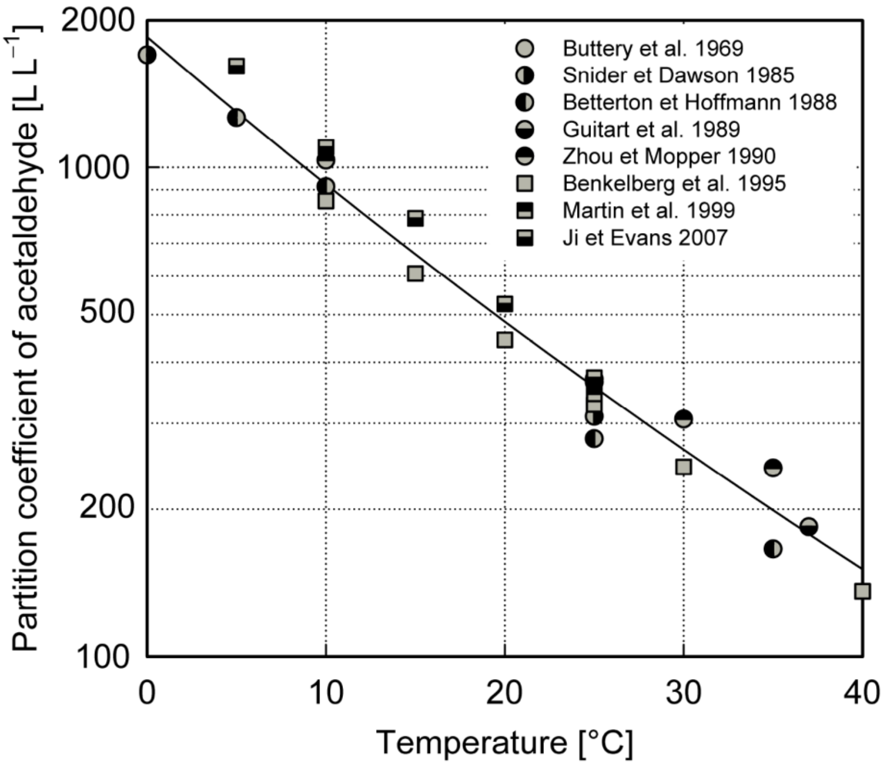

Figure S2.3. Partition coefficients of acetaldehyde in the water/air system taken from the literature and fitted with Equation (S2.5) resulting in the compound-specific constants given in Table S2.1.

References

Aprea, E., Biasioli, F., Märk, T. D., Gasperi, F., PTR-MS study of esters in water and water/ethanol solutions: Fragmentation patterns and partition coefficients. *Int. J. Mass Spectrom.* 2007, *262*, 114–121.

Benkelberg, H.-J., Hamm, S., Warneck, P., Henry's law coefficients for aqueous solutions of acetone, acetaldehyde and acetonitrile, and equilibrium constants for the addition compounds of acetone and acetaldehyde with bisulfite. *J. Atmos. Chem.* 1995, 20, 17–34.

Betterton, E. A., Hoffmann, M. R., Henry's law constants of some environmentally important aldehydes. *Environ. Sci. Technol.* 1988, *22*, 1415–1418.

Brockbank, S. A., *Aqueous Henry's law constants, infinite dilution activity coefficients, and water solubility: Critically evaluated database, experimental analysis, and prediction methods*. PhD thesis, Brigham Young University, Provo Utah 2013.

Butler, J. A. V., Ramchandani, C. N., The solubility of nonelectrolytes. Part II. The influence of the polar group on the free energy of hydration of aliphatic compounds. *J. Chem. Soc.* 1935, 952–955.

Buttery, R. G., Ling, L. C., Guadagni, D. G., Volatilities of aldehydes, ketones, and esters in dilute water solution. *J. Agric. Food Chem.* 1969, *17*, 385–389.

Fenclová, D., Blahut, A., Vrbka, P., Dohnal, V., Böhme, A., Temperature dependence of limiting activity coefficients, Henry's law constants, and related infinite dilution properties of C4-C6 isomeric n-alkyl ethanoates/ethyl n-alkanoates in water. Measurement, critical compilation, correlation, and recommended data. *Fluid Phase Equilib.* 2014, *375*, 347–359.

Guitart, R., Puigdemont, F., Arboix, M., Rapid headspace gas chromatographic method for the determination of liquid/gas partition coefficients. *J. Chromatogr.* 1989, *491*, 271–280.

Grosskopf, K., Die Atemalkohol-Bestimmung als analytische Aufgabe. *Chem. Ztg.* 1954, *78*, 351–356.

Harger, R, N., Raney, B. B., Bridwell, E. G., Kitchel, M. F., The partition ratio of alcohol between air and water, urine and blood; estimation and identification of alcohol in these liquids from analysis of air equilibrated with them. *J. Biol. Chem.* 1950, *183*, 197–213.

Ji, C., Evans, E. M., Using an internal standard method to determine Henry’s law constants. *Environ. Toxicol. Chem.* 2007, *26*, 231–236.

Jones, A. W., Determination of liquid/air partition coefficients for dilute solutions of ethanol in water, whole blood, and plasma. *J. Anal. Toxicol.* 1983, *7*, 193–197.

Kaneko, T., Wang, P.-Y., Sato, A., Partition coefficients of some acetate esters and alcohols in water, blood, olive oil, and rat tissues. *Occup. Environ. Med.* 1994, *51*, 68–72.

Kieckbusch, T. G., King, C. J., An improved method of determining vapor-liquid equilibria for dilute organics in aqueous solution. *J. Chromatogr. Sci.* 1979, *17*, 273–276.

Kolb, B., Welter, C., Bichler, C., Determination of partition coefficients by automatic equilibrium headspace gas chromatography by vapor phase calibration. *Chromatographia* 1992, *34*, 235–240.

Kutsuna, S., Chen, L., Abe, T., Mizukado, J., Uchimaru, T., Tokuhashi, K., Sekiya, A., Henry’s law constants of 2,2,2-trifluoroethyl formate, ethyl trifluoroacetate, and non-fluorinated analogous esters. *Atmos. Environ.* 2005, *39*, 5884–5892.

Löser, C., Urit, T., Nehl, F., Bley, T., Screening of *Kluyveromyces* strains for the production of ethyl acetate: Design and evaluation of a cultivation system. *Eng. Life Sci.* 2011, *11*, 369–381.

Martin, M., Beak, I., Taylor, A. J., Volatile release from aqueous solutions under dynamic headspace dilution conditions. *J. Agric. Food Chem.* 1999, *47*, 4750–4755.

Meylan, W. M., Howard, P. H., Bond contribution method for estimating Henry's law constants. *Environ. Toxicol. Chem.* 1991, *10*, 1283–1291.

Nelson, P. E., Hoff, J. E., Food volatiles: Gas chromatographic determination of partition coefficients in water-lipid systems. *Int. J. Mass Spectrom.* 1968, *228*, 479-482.

Sander, R., Compilation of Henry’s law constants (version 4.0) for water as solvent. *Atmos. Chem. Phys.* 2015, *15*, 4399–4981.

Snider, J. R., Dawson, G. A., Tropospheric light alcohols, carbonyls, and acetonitrile: Concentrations in the southwestern United States and Henry's law data. *J. Geophys. Res.* 1985, *90*, 3797–3805.

Sithersingh, M., *Determination of polar solvents by static headspace extraction – gas chromatography (SHE-GC)*. PhD thesis, Seton Hall University, South Orange NJ 2018.

Urit, T., Löser, C., Wunderlich, M., Bley, T., Formation of ethyl acetate by *Kluyveromyces marxianus* on whey: Studies of the ester stripping. *Bioprocess Biosyst. Eng.* 2011, *34*, 547–559.

Zhou, X., Mopper, K., Apparent partition coefficients of 15 carbonyl compounds between air and seawater and between air and freshwater; implications for air-sea exchange. *Environ. Sci. Technol.* 1990, 24, 1864–1869.

Supporting Information 3: Estimation of parameter β_{VOC}

A new approach for balancing the microbial synthesis of ethyl acetate and other volatile metabolites during aerobic bioreactor cultivations

Christian Löser, Christian Kupsch, Thomas Walther, Andreas Hoffmann

Used symbols are listed in the main part of the work or are explained here in the text.

Parameter β_{VOC} is the efficiency of the condenser for VOC retention. The gas flow absorbs some water when passing through the culture medium in the bioreactor. The moist gas is then flowing through the exhaust-gas condenser where part of this water is condensed and runs back to the bioreactor. This condensate flow of pure water absorbs some gaseous VOC and transports the dissolved VOC back to the reactor. Parameter β_{VOC} represents the amount of the considered VOC which is transported back to the bioreactor, related to the amount of VOC which enters the condenser with the gas flow leaving the headspace of the bioreactor (Fig. S3.1).

Parameter β_{VOC} was determined for three considered VOCs by abiotic stripping tests in the bioreactor system which was used for cultivation experiments described in the main text. The process conditions were identical in the stripping and cultivation experiments.

According to the above-given definition, parameter β_{VOC} ranges from zero (no retention of the VOC) to one (complete retention). The larger β_{VOC} is, the more intensive the VOC retention and the slower the stripping of VOC are. The stripping experiments clearly demonstrated that β_{VOC} highly depends on the studied VOC.

Here, a model equation is derived which allows the estimation of β_{VOC}. This equation also discloses the variables which take influence on β_{VOC}. This modeling is based on balancing the VOC in the condenser.

Balancing of the VOC in the condenser of a bioreactor

The following scheme depicts relevant local positions and mass flows of the transported VOC.

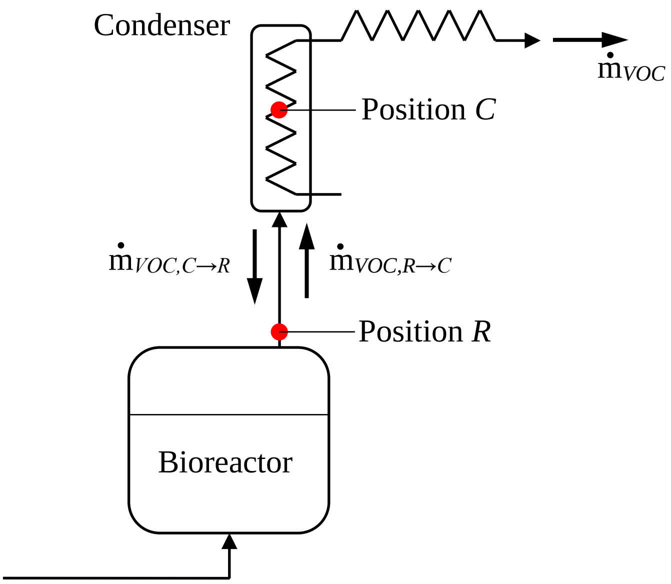

Figure S3.1. Scheme of the bioreactor with condenser for visualization of the mass flows of VOC and for representation of local positions

According to the definition of β_{VOC}, the following equation is valid:

$$\beta_{VOC} = \frac{\dot{m}_{VOC,C\rightarrow R}}{\dot{m}_{VOC,R\rightarrow C}} \tag{S3.1}$$

The mass flow of VOC from the condenser back to the bioreactor is a condensate-borne flow, i. e., it is the VOC which dissolves in water being condensed by cooling and returned to the bioreactor. This mass flow is expressed as follows:

$$\dot{m}_{VOC,C\rightarrow R} = F_{L,C} \cdot C_{VOC,L,C} \tag{S3.2}$$

Herein, $F_{L,C}$ is the condensate flow back to the reactor, and $C_{VOC,L,C}$ is the VOC concentration in this condensate. The condensate flow $F_{L,C}$ depends on several factors such as the flow and water content of the exhaust gas, and the dew-point temperature in the condenser. Substitution of the condensate-borne mass flow in Equation (S3.1) by Equation (S3.2) results in:

$$\beta_{VOC} = \frac{F_{L,C}}{\dot{m}_{VOC,R\rightarrow C}} \cdot C_{VOC,L,C} \tag{S3.3}$$

The VOC concentration in the condensate $C_{VOC,L,C}$ depends on the VOC concentration in the gas phase within the condenser $C_{VOC,G,C}$. The higher $C_{VOC,G,C}$ is, the higher $C_{VOC,L,C}$ is. Assuming an equilibrium between the condensate and the surrounding gas, the following equation is true:

$$C_{VOC,L,C} = K_{VOC,L/G}^{water}(T_C) \cdot C_{VOC,G,C} \tag{S3.4}$$

Parameter $K_{VOC,L/G}^{water}(T_C)$ is the partition coefficient for the considered VOC in a water/air system at the temperature T_C. The gas is here in contact with the condensate, i. e., with pure water. Substitution of the liquid concentration in Equation (S3.3) by Equation (S3.4) gives:

$$\beta_{VOC} = \frac{F_{L,C} \cdot K_{VOC,L/G}^{water}(T_C)}{\dot{m}_{VOC,R\rightarrow C}} \cdot C_{VOC,G,C} \tag{S3.5}$$

The VOC concentration $C_{VOC,G,C}$ is not identical with the VOC concentration $C_{VOC,G,R}$ at the inlet of the condenser. The difference is caused by the VOC transfer from the gas to the condensate as well as by some volume effects (cooling and dehumidification diminishes the gas flow and thus increases the VOC concentration). The unknown $C_{VOC,G,C}$ concentration is described as the relation between the gas-borne VOC mass flow leaving the condenser ($\dot{m}_{VOC,G}$) and the gas flow within the condenser:

$$C_{VOC,G,C} = \frac{\dot{m}_{VOC,G}}{F_{G,C}} \tag{S3.6}$$

The mass flow $\dot{m}_{VOC,G}$ in Equation (S3.6) is expressed as the mass flow of VOC entering the condenser minus the mass flow of the dissolved VOC returning to the reactor:

$$\dot{m}_{VOC,G} = \dot{m}_{VOC,R\rightarrow C} - \dot{m}_{VOC,C\rightarrow R} \tag{S3.7}$$

Substitution of $\dot{m}_{VOC,C\rightarrow R}$ in Equation (S3.7) by Equation (S3.1) yields:

$$\dot{m}_{VOC,G} = \dot{m}_{VOC,R\rightarrow C} \cdot (1 - \beta_{VOC}) \tag{S3.8}$$

Combination of Equations (S3.5), (S3.6) and (S3.8) is aimed at elimination of $C_{VOC,G,C}$ and yields:

$$\beta_{VOC} = \frac{F_{L,C} \cdot K_{VOC,L/G}^{water}(T_C)}{\dot{m}_{VOC,R\rightarrow C}} \cdot \frac{\dot{m}_{VOC,R\rightarrow C} \cdot (1 - \beta_{VOC})}{F_{G,C}} \tag{S3.9}$$

Rearrangement of Equation (S3.9) for separating β_{VOC} results in:

$$\beta_{VOC} = \frac{1}{\frac{F_{G,C}}{F_{L,C} \cdot K_{VOC,L/G}^{water}(T_C)} + 1} \tag{S3.10}$$

Equation (S3.10) allows to evaluate the influence of condenser parameters on β_{VOC} at least in a qualitative way. A large $F_{L,C}$-$F_{G,C}$ ratio and a high $K_{VOC,L/G}^{water}(T_C)$ coefficient let β_{VOC} approach to one while a small $F_{L,C}$-$F_{G,C}$ ratio and a low $K_{VOC,L/G}^{water}(T_C)$ coefficient results in a β_{VOC} value being nearly zero. However, the $F_{L,C}$-$F_{G,C}$ ratio is not subject of high variation in the case of a well-working condenser since an increasing $F_{G,C}$ value gives a higher condensate flow $F_{L,C}$, i. e., the $F_{L,C}$-$F_{G,C}$ ratio is nearly constant for a given condenser and a fixed dew-point temperature.

The condensate flow is calculable by balancing the condensation process for the cooled gas flow:

$$F_{L,C} = \frac{\dot{m}_{W,C\to R}}{\varrho_{W,L}} = \frac{\dot{m}_{W,R\to C} - \dot{m}_{W,G}}{\varrho_{W,L}} \tag{S3.11}$$

The $\dot{m}_{W,R\to C}$ flow is the mass of water entering the condenser with the moist gas per hour, while the $\dot{m}_{W,G}$ flow represents the mass of water leaving the condenser with the cooled gas. And $\varrho_{W,L}$ is the density of liquid water. Both mass flows are expressed as gas flows multiplied by the respective volumetric water content and density of gaseous water:

$$F_{L,C} = \frac{F_{G,R} \cdot x_{W,G,R} \cdot \varrho_{W,G,R} - F_G \cdot x_{W,G} \cdot \varrho_{W,G}}{\varrho_{W,L}} \tag{S3.12}$$

The densities of gaseous water only depend on temperature (assuming that the gaseous water acts as an ideal gas und that the process proceeds at ambient pressure) and are substituted:

$$F_{L,C} = F_G \cdot \frac{\varrho_{W,G}^0}{\varrho_{W,L}} \cdot \left(\frac{F_{G,R}}{F_G} \cdot \frac{T_0}{T_{G,R}} \cdot x_{W,G,R} - \frac{T_0}{T_G} \cdot x_{W,G} \right) \tag{S3.13}$$

Herein, $\varrho_{W,G}^0$ stands for the density of gaseous water at standard conditions, i. e., at 101325 Pa and 273.15 K. Substitution of the condensate flow in Equation (S3.10) by using Equation (S3.13) gives:

$$\beta_{VOC} = \frac{1}{\frac{F_{G,C}}{F_G \cdot \frac{\varrho_{W,G}^0}{\varrho_{W,L}} \cdot \left(\frac{F_{G,R}}{F_G} \cdot \frac{T_0}{T_{G,R}} \cdot x_{W,G,R} - \frac{T_0}{T_G} \cdot x_{W,G} \right) \cdot K_{VOC,L/G}^{water}(T_C)} + 1} \tag{S3.14}$$

The gas flow $F_{G,C}$ is expressed with the gas flow at the system outlet, $F_{G,C} = F_G \cdot T_C / T_G$, yielding:

$$\beta_{VOC} = \frac{1}{\frac{T_C}{T_0 \cdot \frac{\varrho_{W,G}^0}{\varrho_{W,L}} \cdot \left(\frac{F_{G,R}}{F_G} \cdot \frac{T_G}{T_{G,R}} \cdot x_{W,G,R} - x_{W,G} \right) \cdot K_{VOV,L/G}^{water}(T_C)} + 1} \tag{S3.15}$$

Substitution of the $F_{G,R}$-F_G ratio by Equation (7) of the main text and rearrangement results in:

$$\beta_{VOC} = \frac{1}{\frac{T_C}{T_0} \cdot \frac{\varrho_{W,L}}{\varrho_{W,G}^0} \cdot \frac{1 - x_{W,G,R}}{x_{W,G,R} - x_{W,G}} \cdot \frac{1}{K_{VOC,L/G}^{water}(T_C)} + 1} \tag{S3.16}$$

This means that β_{VOC} is defined by T_0 = 273.15 K, T_C as the temperature at the phase equilibrium in the condenser, $\varrho_{W,G}^0$ = 0.8038 g L^{-1}, $\varrho_{W,L} \approx$ 1000 g L^{-1}, $x_{W,G,R}$ = 0.0469 L L^{-1} at a reactor temperature

of 32 °C, $x_{W,G}$ = 0.0143 L L^{-1} at the condenser-outlet temperature of 12.5 °C, and by $K_{VOC,L/G}^{water}(T_C)$ as the partition coefficient of the VOC in a water/air system at equilibrium temperature T_C.

According to Equation (S3.16), the gas flow does obviously not influence β_{VOC}, apart from very high gas flows, where the assumption of near-equilibrium conditions is no longer fulfilled and the condenser-outlet temperature shifts to higher values. This finding is not surprising since an increase in the gas flow heightens the VOC discharge from the reactor but also increases the return transport of the VOC dissolved in the more intensely formed condensate. Thus, variation of the gas flow does not change the ratio between the two VOC mass flows.

Parameter T_C is the temperature at which the phase equilibrium is established between the gas flow and condensate flow. The temperature of the gas phase passing the condenser diminishes from 32 °C at the condenser inlet (T_R) down to 12.5 °C at the condenser outlet. The equilibration temperature T_C must thus lie between these two temperatures. Water that condenses near the outlet and absorbs a larger amount of VOC runs down to the bioreactor, whereby it is warmed up again to some degree and is subjected to exchange processes with the gas flow. These exchange processes include the re-transport of already absorbed VOC back to the gas phase. The temperature T_C is assumed to be distinctly larger than the minimum temperature at the condenser outlet.

Temperature T_C influences parameter β_{VOC} in two ways, namely directly as a variable in Equation (S3.16) and indirectly via the temperature dependency of the partition coefficient. The direct effect is small since variation of T_C between 12.5 and 32 °C (the possible minimum and maximum values) results in only a slight change of β_{VOC}; model calculations demonstrated, that the just mentioned T_C variation in Equation (S3.16) let the parameter β_{VOC} vary only by ± 3.3% at a maximum (provided that the partition coefficient is not smaller than 50 L L^{-1}). The indirect effect of T_C on β_{VOC} via $K_{VOV,L/G}^{water}(T_C)$ is, however, much more important since partition coefficients of VOCs highly depend on temperature (for details see Supporting Information 2) so that the selected temperature T_C substantially influences the calculated β_{VOC} value.

$K_{VOV,L/G}^{water}(T)$ for ethyl acetate, ethanol and acetaldehyde

The estimation of parameter β_{VOC} requires temperature-dependent partition coefficients for ethyl acetate, ethanol and acetaldehyde in a water/air system. In Supporting Information 2, these $K_{VOV,L/G}^{water}(T)$ functions were derived from literature data in a temperature range from 0 to 40 °C. These three $K_{VOV,L/G}^{water}(T)$ dependencies are depicted in Figure S3.2 to demonstrate the high influence of the temperature on the partition coefficient. A change of temperature by 40 K alters the partition coefficient by a factor of 12 to 18, depending on the considered VOC.

The $K_{VOV,L/G}^{water}(T)$ dependencies were used to calculate temperature-dependent $\beta_{VOC}(T_C)$ functions by Equation (S3.16), depicted in Figure S3.3. The higher the assumed equilibration temperature T_C is, the smaller the β_{VOC} parameters become. All studied compounds exhibit the same qualitative but different quantitative $\beta_{VOC}(T_C)$ pattern. The calculated retention of ethanol is highest, the retention of acetaldehyde is smaller but relevant, and the retention of ethyl acetate is almost negligible. This result clearly corresponds with the observations in the abiotic stripping experiments. The β_{VOC} values calculated for an equilibrium temperature of T_C = 20 °C are similar to the measured β_{VOC} values.

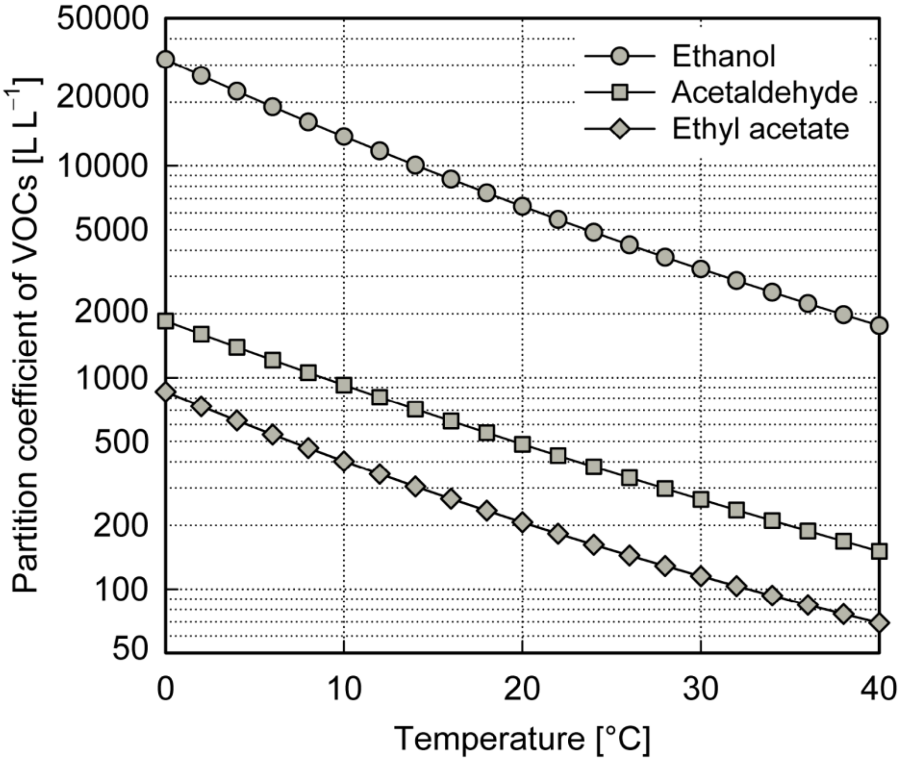

Figure S3.2. Partition coefficients of ethyl acetate, ethanol and acetaldehyde depending on temperature in water/air systems (originating from literature data and taken from Supporting Information 2)

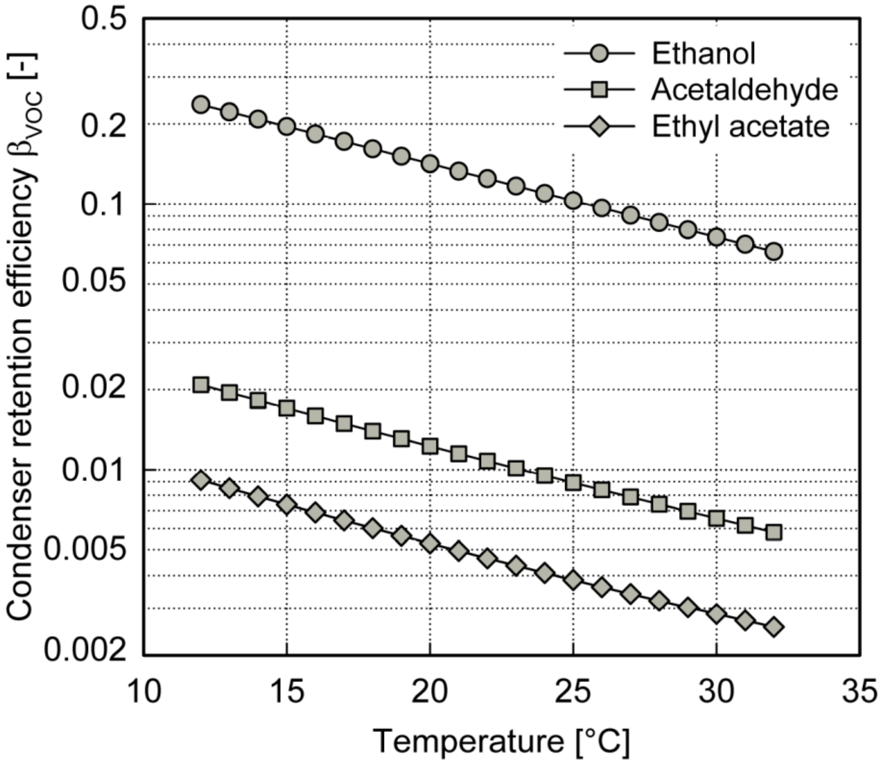

Figure S3.3. VOC retention efficiency of the condenser β_{VOC} depending on the assumed equilibration temperature T_C calculated by Equation (S3.16), using the $K_{VOV,L/G}^{water}(T)$ values from Figure S3.2 for the range of 12.5 to 32 °C (representing the possible minimum and maximum of T_C)

Supporting Information 4: Combined stripping of VOCs

A new approach for balancing the microbial synthesis of ethyl acetate and other volatile metabolites during aerobic bioreactor cultivations

Christian Löser, Christian Kupsch, Thomas Walther, Andreas Hoffmann

Used symbols are listed in the main part of the work or are explained here in the text.

The new method for balancing the microbial synthesis of ethyl acetate and other volatile metabolites during aerobic bioreactor cultivations is solely based on repeatedly measured gas-phase concentrations of the VOCs of interest. The liquid-phase VOC concentrations, required for the balancing, are not measured but derived from the gas-phase VOC concentrations assuming a phase equilibrium between the culture medium and the headspace gas in the bioreactor. This assumption is true as long as the phase-transfer coefficient of the considered VOC, $k_{VOC,L}a$, is distinctly larger than the $(F_{G,R}/V_L)/K_{VOC,L/G}$ expression (for details see Chapter 3.1 of the main text).

Calculation of the liquid-phase concentration of a VOC ($C_{VOC,L}$) from the measured gas-phase concentration of the same VOC ($C_{VOC,G}$) is based on the following equation (for details it is referred to Supporting Information 1):

$$C_{VOC,L} = C_{VOC,G} \cdot \frac{F_G}{F_{G,R}} \cdot \frac{K_{VOC,L/G}}{1 - \beta_{VOC}} \qquad \text{(S4.1)}$$

This calculation requires the two parameters $K_{VOC,L/G}$ and β_{VOC}. $K_{VOC,L/G}$ is the partition coefficient of the considered VOC and β_{VOC} is the retention efficiency of this VOC by the exhaust-gas condenser. These two parameters can be determined by two stripping experiments: one stripping test is performed with a condenser while the other stripping test is done without a condenser.

Such stripping experiments were performed separately for the three volatiles ethyl acetate, ethanol and acetaldehyde which was quite time-consuming. Actually, according to Eq. (S4.1) only the $K_{VOC,L/G}/(1-\beta_{VOC})$ term must be known for each VOC to enable the calculation of $C_{VOC,L}$ from $C_{VOC,G}$. The needed term is determinable by stripping experiments using the bioreactor fitted with the condenser. According to Eq. (16) in the main text, the wanted term is obtained from the stripping rate and the applied specific gas-flow rate: $K_{VOC,L/G}/(1-\beta_{VOC}) = -(F_{G,R}/V_L)/k_{VOC}$.

The expenditure of human labor can be even more reduced when the stripping of several VOCs is combined in only one experiment. However, the stripping rate of one VOC could be influenced by the presence of another VOC. For example, Aprea et al. [2007] and Ammari et Schroen [2019] observed that the presence of ethanol in the aqueous solution of ethyl acetate increases the partition coefficient $K_{EA,L/G}$ (ethanol obviously heightens the ester solubility in water) which would decrease the stripping rate and seemingly increase the $K_{VOC,L/G}/(1-\beta_{VOC})$ term. The applied initial VOC concentrations must therefore be low enough to avoid such interferences between several stripped VOCs (e. g., ≤ 1 g L^{-1} in the liquid phase).

For proving the correctness of this proposed simplification of $K_{VOC,L/G}/(1-\beta_{VOC})$ determination for several VOCs in one experiment, the stripping of the three volatiles of interest was studied as described in the main text by using the bioreactor with the exhaust-gas condenser. All experimental parameters were identical with the only difference that ethyl acetate, ethanol and acetaldehyde were combined in one cultivation medium. As mentioned above, the initial liquid-phase concentrations should be low and amounted to 0.88 g L^{-1} for ethyl acetate, 1.13 g L^{-1} for ethanol and 0.67 g L^{-1} for acetaldehyde (determined from the initial gas concentrations via Eq. (S4.1)).

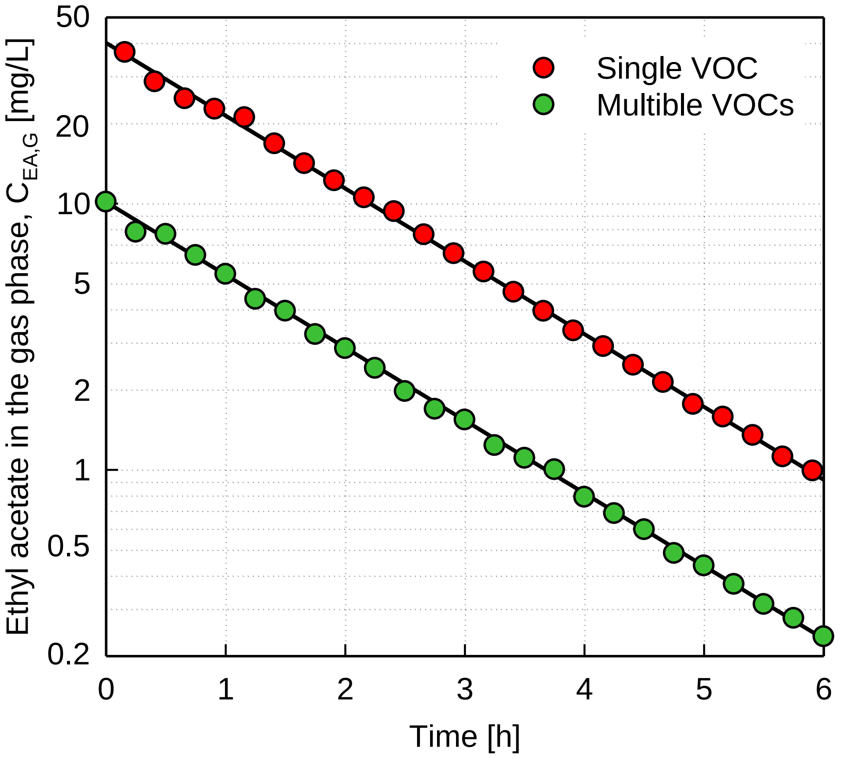

Figure S4.1. Concentration of ethyl acetate in the gas phase during stripping from a 1-L stirred bioreactor using 0.6 L glucose-based mineral medium (stirring with 1200 rpm at 32 °C and aeration with 30 L/h at standard conditions); Stripping was performed without a condenser and with an initial liquid-phase concentration of about 3 g/L ethyl acetate as a single compound (red symbols) or with a condenser and a mixture of ethyl acetate, ethanol and acetaldehyde each present with an initial concentration of about 0.9 g/L in the liquid phase (green symbols).

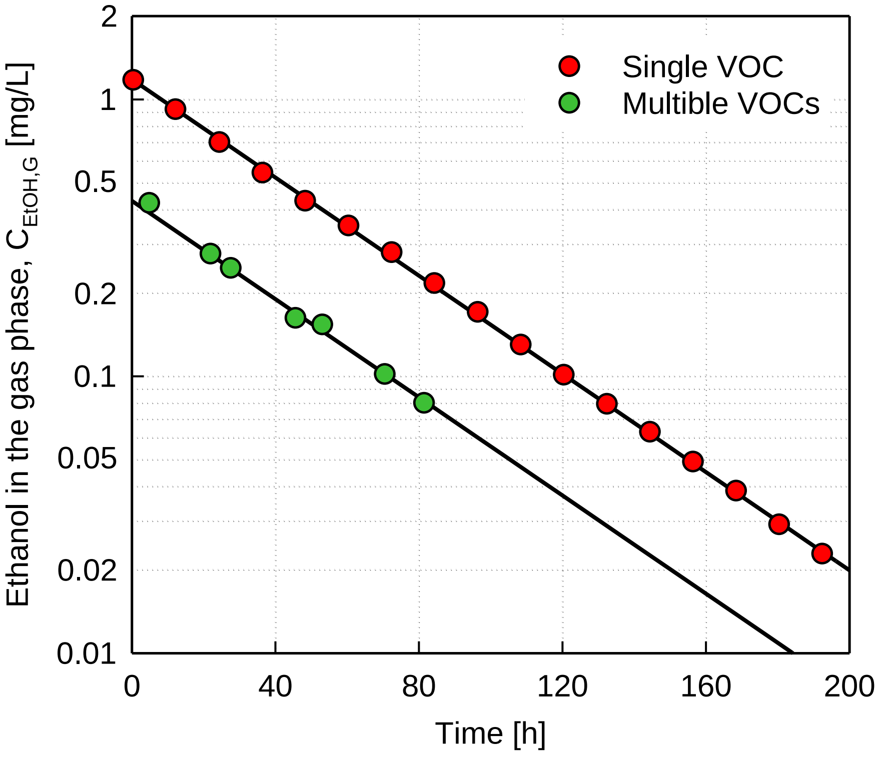

Figure S4.2. Concentration of ethanol in the gas phase during stripping from a 1-L stirred bioreactor fitted with a condenser using 0.6 L glucose-based mineral medium (stirring with 1200 rpm at 32 °C and aeration with 30 L/h at standard conditions); Stripping was performed with an initial liquid-phase concentration of about 3 g/L ethanol as a single compound (red symbols) or with a mixture of ethyl acetate, ethanol and acetaldehyde each present with an initial concentration of about 0.9 g/L in the liquid phase (green symbols).

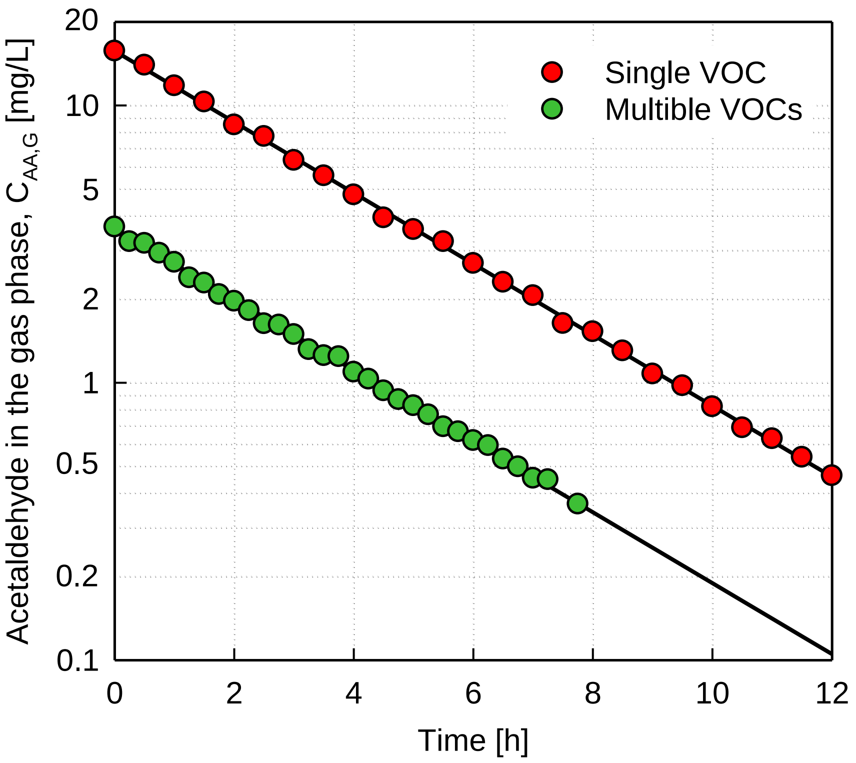

Figure S4.3. Concentration of acetaldehyde in the gas phase during stripping from a 1-L stirred bioreactor fitted with a condenser using 0.6 L glucose-based mineral medium (stirring with 1200 rpm at 32 °C and aeration with 30 L/h at standard conditions); Stripping was performed with an initial liquid-phase concentration of about 3 g/L acetaldehyde as a single compound (red symbols) or with a mixture of ethyl acetate, ethanol and acetaldehyde each contained with an initial concentration of about 0.9 g/L in the liquid phase (green symbols).

The figures present the results of the combined stripping experiment for ethyl acetate (Fig. S4.1), ethanol (Fig. S4.2) and acetaldehyde (Fig. S4.3) together with the results which were obtained in stripping experiments with a single compound (data taken from Fig. 1 of the main text). There was no distinction between the stripping behavior, and the stripping rates were identical independently if the stripping was determined with a single compound or with the mixture of the three tested compounds. This means that the stripping rates of several compounds are determinable in only one stripping experiment. In this way, the experimental effort can be significantly reduced.

References

Ammari, A., Schroen, K., Effect of ethanol and temperature on partition coefficients of ethyl acetate, isoamyl acetate, and isoamyl alcohol: Instrumental and predictive investigation. *J. Chem. Eng. Data* 2019, *64*, 3224–3230.

Aprea, E., Biasioli, F., Märk, T. D., Gasperi, F., PTR-MS study of esters in water and water/ethanol solutions: Fragmentation patterns and partition coefficients. *Int. J. Mass Spectrom.* 2007, *262*, 114–121.

Supporting Information 5: $K_{EA,L/G}$ variation during processes

A new approach for balancing the microbial synthesis of ethyl acetate and other volatile metabolites during aerobic bioreactor cultivations

Christian Löser, Christian Kupsch, Thomas Walther, Andreas Hoffmann

Used symbols are listed in the main part of the work or are explained here in the text.

A new method for balancing the microbial synthesis of ethyl acetate and other volatiles in bioreactors were developed where the liquid-phase concentrations of VOCs are calculated from their measured gas-phase concentrations by using the following equation (for details see main text and Supporting Information 1):

$$C_{VOC,L} = C_{VOC,G} \cdot \frac{T_G}{T_{G,R}} \cdot \frac{1 - x_{W,G,R}}{1 - x_{W,G}} \cdot \frac{K_{VOC,L/G}}{1 - \beta_{VOC}} \qquad \text{(S5.1)}$$

$C_{VOC,L}$ and $C_{VOC,G}$ exhibit a constant relation as long as all parameters of Eq. S5.1 remain constant during the cultivation process. This is true in any case for the water content of the gas phases $x_{W,G,R}$ and $x_{W,G}$, the temperatures T_G and $T_{G,R}$, and for the retention efficiency β_{VOC}. The water content $x_{W,G,R}$ is determined by temperature T_R, the water content $x_{W,G}$ depends on the temperature at the exit of the exhaust-gas condenser, and all temperatures including T_G and $T_{G,R}$ did not change during the cultivation process. The retention efficiency of the condenser for the considered VOC, β_{VOC}, is determined by the condensation process in the exhaust-gas condenser and stayed also constant during the whole process.

The partition coefficient $K_{VOC,L/G}$ requires closer examination since this parameter depends on the properties of the cultivation medium which eventually change during the process. This especially applies to the sugar concentration which is high at the beginning and becomes zero after sugar depletion. And the content of minerals increases due to dosage of 2 M NaOH for pH correction. Both, the temporally decreasing sugar content [Covarrubias-Cervantes et al. 2004; Covarrubias-Cervantes et al. 2005; Urit et al. 2011] and the gradually increasing mineral content [Jones 1983; Zhou et Mopper 1990; Benkelberg et al. 1995; Al-Sahhaf et al. 1999] can change the partition coefficient.

Even the growing biomass could take some influence on $K_{VOC,L/G}$ since the VOCs are dissolved in the aqueous phase but not in the suspension. The rising cell concentration results in an increasing discrepancy between the aqueous-phase volume and the suspension volume [Löser et al. 2018]. However, this effect becomes only relevant at high cell densities. In the considered case, the biomass concentration did not exceed a value of 5 g L^{-1} so that the effect of the grown biomass on the partition coefficient is negligible.

The figures shown below are presented to demonstrate that the temporal change of the partition coefficient was only marginal in previous bioreactor cultivations of *Kluyveromyces marxianus* DSM 5422 in spite of significant changes of the sugar and mineral content of the cultivation medium.

The original data for preparing Figs. 5.1 to 5.3 originate from Löser et al. [2013]. These diagrams present measured liquid-phase and gas-phase concentrations of ethyl acetate which was formed from sugar during the batch cultivation of *K. marxianus* DSM 5422 in a 70-L stirred reactor. The used DW basic medium contained 75 g L^{-1} sugar which was completely consumed during the process. 5 M KOH was added by a controller to ensure pH ≥ 5. The KOH addition resulted in an increase of the mineral content in the range from 3.8 to 5.6 g L^{-1} K^+ ions.

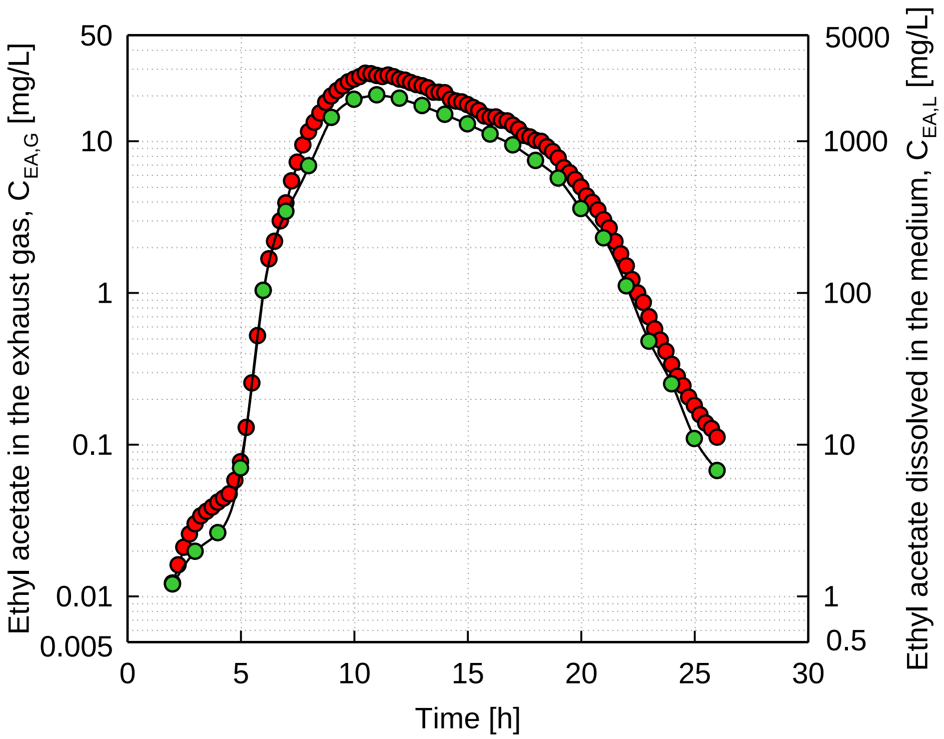

Figure S5.1. Measured concentrations of ethyl acetate in the exhaust gas (red symbols) and culture medium (green symbols) during the aerobic batch cultivation of *K. marxianus* DSM 5422 in a 70-L stirred bioreactor; Cultivation in 38.2 L whey-borne DW basic medium supplemented with an iron-free trace-element solution and inoculated with 0.5 L pre-culture, pH controlled to ≥ 5, bioreactor stirred with 800 rpm at 32 °C, and aerated with 3,000 L h^{-1} at standard conditions; the total initial iron content amounted to 53 µg L^{-1} (sum of iron dissolved in the medium and contained in the biomass of inoculum); Data taken from [Löser et al. 2013].

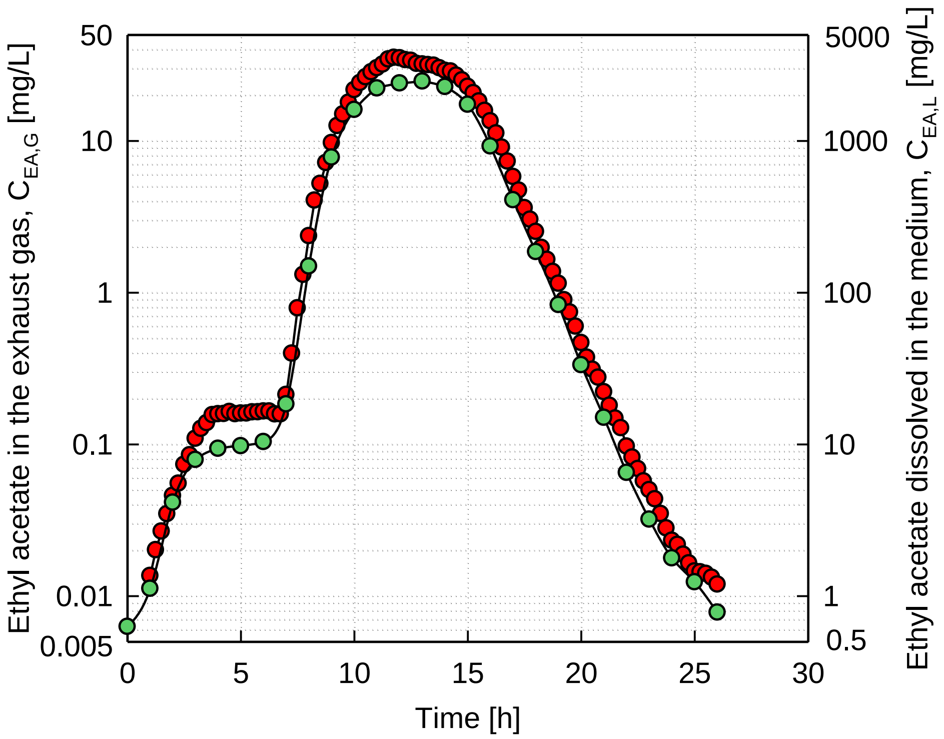

Figure S5.2. Measured concentrations of ethyl acetate in the exhaust gas (red symbols) and culture medium (green symbols) during the aerobic batch cultivation of *K. marxianus* DSM 5422 in a 70-L stirred bioreactor; Cultivation in 38.2 L whey-borne DW basic medium supplemented with an iron-free trace-element solution and inoculated with 0.5 L pre-culture, pH controlled to ≥ 5, bioreactor stirred with 800 rpm at 32 °C, and aerated with 3,000 L h^{-1} at standard conditions; the total initial iron content amounted to 79 µg L^{-1} (sum of iron dissolved in the medium and contained in the biomass of inoculum); Data taken from [Löser et al. 2013].

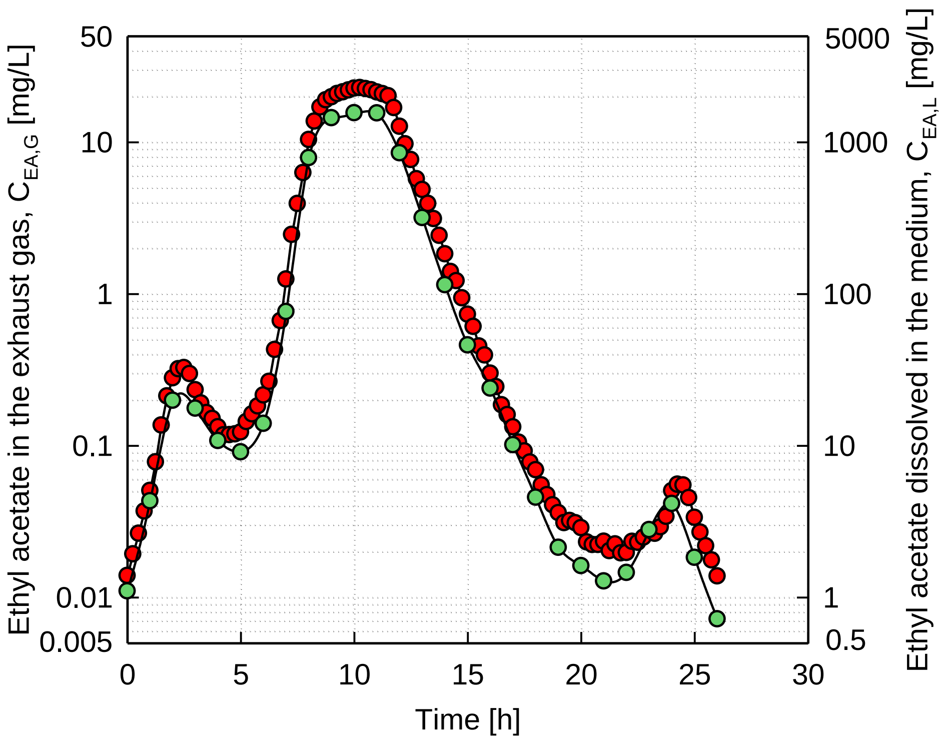

Figure S5.3. Measured concentrations of ethyl acetate in the exhaust gas (red symbols) and culture medium (green symbols) during the aerobic batch cultivation of *K. marxianus* DSM 5422 in a 70-L stirred bioreactor; Cultivation in 38.2 L whey-borne DW basic medium supplemented with an iron-free trace-element solution and inoculated with 0.5 L pre-culture, pH controlled to ≥ 5, bioreactor stirred with 800 rpm at 32 °C, and aerated with 3,000 L h^{-1} at standard conditions; the total initial iron content amounted to 182 µg L^{-1} (sum of iron dissolved in the medium and contained in the biomass of inoculum); Data taken from [Löser et al. 2013].

Figs. S5.1 to S5.3 represent the concentrations of ethyl acetate in the gas as well as the liquid phase over time in a logarithmic scaling during aerobic cultivation of *K. marxianus* DSM 5422 in a 70-L stirred bioreactor in DW basic medium under iron limitation (for details see [Löser et al. 2013]). The only varied parameter was the iron content of the inoculum. The logarithmic $C_{EA,G}(t)$ and $C_{EA,L}(t)$ curves are very similar: the concentrations at first increased due to a rising synthesis rate of ethyl acetate, then exhibited a plateau over a certain time period (synthesis of ethyl acetate in this period occurred with a high and less varying rate), and then linearly decreased because of the ester stripping.

The constant geometric distance between the $C_{EA,G}(t)$ and $C_{EA,L}(t)$ curves over the whole process is explainable by a constant relation between both depicted concentrations. This constant $C_{EA,G}(t)$-$C_{EA,L}(t)$ ratio argues for a nearly unchanged partition coefficient over time ($K_{EA,L/G} \approx 78$ L L^{-1}) although the sugar concentration as well as the mineral content of the medium distinctly changed during the process.

In Fig. S5.4, the partition coefficient is directly presented for three similar processes. The data were obtained during aerobic batch cultivations of *K. marxianus* DSM 5422 in a 1-L stirred reactor under iron limitation at various temperature regimes (for details see [Urit et al. 2013]). The cultivation occurred at 32 °C, at 42 °C, or started at 32 °C and was later suddenly heightened to 42 °C. The used DW basic medium contained 78 g L^{-1} sugar which was completely consumed during the process. The pH was controlled by supply of 2 M KOH which resulted in an increase of the mineral content in the range from 3.0 to 5.0 g L^{-1} K^{+} ions. The partition coefficient was highly influenced by the temperature ($K_{EA,L/G} \approx 77.6$ L L^{-1} at 32 °C, $K_{EA,L/G} = 44.2$ L L^{-1}at 42 °C), and the sudden increase of temperature from 32 to 42 °C let the partition coefficient drop immediately. However, the temporal changes of the sugar and mineral concentrations did not significantly modify the partition coefficient over time.

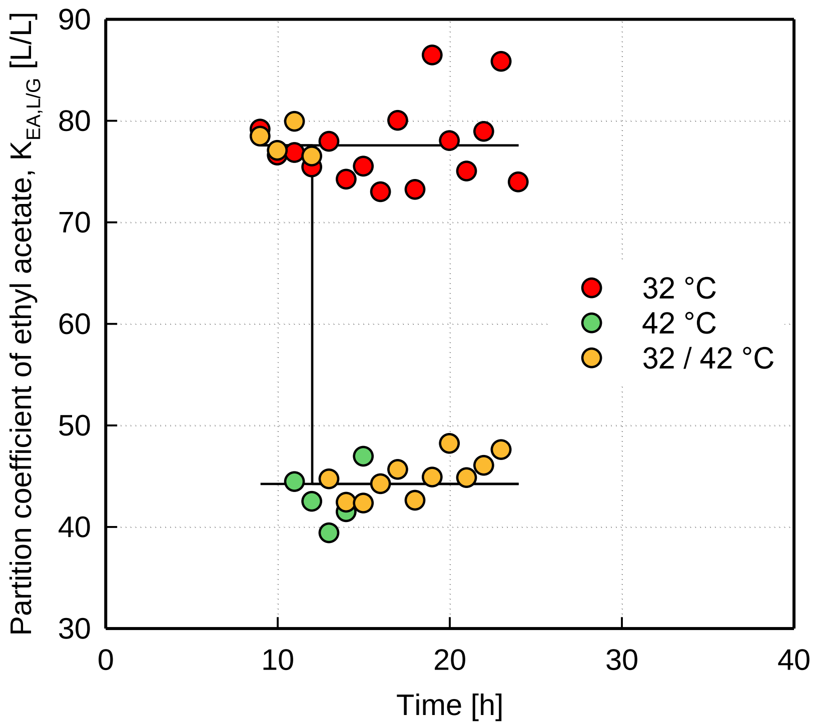

Figure S5.4. Partition coefficient of ethyl acetate in the culture medium/air system in three aerobic batch cultivations of *K. marxianus* DSM 5422 in a 1-L stirred bioreactor at 32 °C (red symbols), at 42 °C (green symbols), or at 32 °C in a 12-h lasting initial phase and then continued at 42 °C (orange symbols); Cultivation in 0.6 L whey-borne DW basic medium without supplementation of trace elements, pH controlled to ≥ 5, bioreactor stirred with 1,200 rpm and gassed with 50 L/h air at standard conditions; Data taken from [Urit et al. 2013].

These data demonstrate, that the temporal change of the partition coefficient was only marginal in DW basic medium although the sugar and mineral content distinctly varied during the process. Cultivation of *K. marxianus* DSM 5422 in glucose-based mineral medium (see experiments described in the main text) resulted in significantly smaller changes of the sugar and mineral content (consumption of 20 g L^{-1} glucose, and dosage of 1.5 or 0.6 g L^{-1} Na^+ ions for pH correction); the expected $K_{EA,L/G}$ variations are thus even lower. The use of temporally constant partition coefficients for the balancing the VOC synthesis based on repeatedly measured gas-phase concentrations is therefore admissible.

References

Al-Sahhaf, T. A., Kapetanovic, E., Kadhem, Q., Salt effects on liquid-liquid equilibria in the partially miscible systems water + 2-butanone and water + ethyl acetate. *Fluid Phase Equilib.* 1999, *157*, 271–283.

Benkelberg, H.-J., Hamm, S., Warneck, P., Henry's law coefficients for aqueous solutions of acetone, acetaldehyde and acetonitrile, and equilibrium constants for the addition compounds of acetone and acetaldehyde with bisulfite. *J. Atmos. Chem.* 1995, *20*, 17–34.

Covarrubias-Cervantes, M., Champion, D., Debeaufort, F., Voilley, A., Aroma volatility from aqueous sucrose solutions at low and subzero temperatures. *J. Agric. Food Chem.* 2004, *52*, 7064–7069.

Covarrubias-Cervantes, M., Bongard, S., Champion, D., Voilley, A., Temperature effect on solubility of aroma compounds in various aqueous solutions. *Lebensm.-Wiss. Technol.* 2005, *38*, 371–378.

Jones, A. W., Determination of liquid/air partition coefficients for dilute solutions of ethanol in water, whole blood, and plasma. *J. Anal. Toxicol.* 1983, *7*, 193–197.

Löser, C., Urit, T., Stukert, A., Bley, T., Formation of ethyl acetate from whey by Kluyveromyces marxianus on a pilot scale. J. Biotechnol. 2013, 163, 17–23.

Urit, T., Löser, C., Wunderlich, M., Bley, T., Formation of ethyl acetate by *Kluyveromyces marxianus* on whey: Studies of the ester stripping. *Bioprocess Biosyst. Eng.* 2011, *34*, 547–559.

Urit, T., Li, M., Bley, T., Löser, C., Growth of *Kluyveromyces marxianus* and formation of ethyl acetate depending on temperature. *Appl. Microbiol. Biotechnol.* 2013, *97*, 10359–10371.

Zhou, X., Mopper, K., Apparent partition coefficients of 15 carbonyl compounds between air and seawater and between air and freshwater; implications for air-sea exchange. *Environ. Sci. Technol.* 1990, *24*, 1864–1869.
